# Supplementary material for: Assessment of the cPAS-based BGISEQ-500 platform for metagenomic sequencing
Source: Gigascience. 2017 Dec 23;7(3):gix133. doi: 10.1093/gigascience/gix133 (PMC5848809; doi:10.1093/gigascience/gix133)
Supplement: GIGA-D-17-00215_Revision_2.pdf [file gix133_giga-d-17-00215_revision_2.pdf]

GigaScience

Assessment of the cPAS-based BGISEQ-500 platform for metagenomic sequencing

--Manuscript Draft--

**Manuscript Number:**	GIGA-D-17-00215R2
**Full Title:**	Assessment of the cPAS-based BGISEQ-500 platform for metagenomic sequencing
**Article Type:**	Data Note

Funding Information:

National Key Research and Development Program of China (No.SQ2017YFSF090209)	Dr. Junhua Li
National Natural Science Foundation of China (No.31601073)	Dr. Junhua Li
Shenzhen Municipal Government of China (No.JSGG20160229172752028)	Dr. Junhua Li
Shenzhen Municipal Government of China Peacock Plan (No. KQTD20150330171505310)	Dr. Wenwei Zhang
American Diabetes Association Pathway Award (Grant #1-17-INI-13)	Dr. Aleksandar David Kostic
Smith Family Foundation Award	Dr. Aleksandar David Kostic
NIH/ NHGRI T32 HG002295 (HG002295)	Dr. Aleksandar David Kostic
NIH/ NIGMS T32 GM074897 (GM074897)	Dr. Aleksandar David Kostic

Abstract:

Background: More extensive use of metagenomic shotgun sequencing in microbiome research relies on the development of high-throughput, cost-effective sequencing. Here we present a comprehensive evaluation of the performance of the new high-throughput sequencing platform BGISEQ-500 for metagenomic shotgun sequencing and compare its performance with that of two Illumina platforms.
Findings: Using fecal samples from 20 healthy individuals we evaluated the intra-platform reproducibility for metagenomic sequencing on the BGISEQ-500 platform in a setup comprising 8 library replicates and 8 sequencing replicates. Cross-platform consistency, was evaluated by comparing 20 pairwise replicates on the BGISEQ-500 platform versus the Illumina HiSeq 2000 platform and the Illumina HiSeq 4000 platform. In addition, we compared the performance of the two Illumina platforms against each other.
By a newly developed overall accuracy quality control method, an average of 82.45 million high quality reads (96.06% of raw reads) per sample with 90.56% of bases scoring Q30 and above was obtained using the BGISEQ-500 platform. Quantitative analyses revealed extremely high reproducibility between BGISEQ-500 intra-platform replicates. Cross-platform replicates differed slightly more than intra-platform replicates, yet a high consistency was observed. Only a low percentage (2.02% - 3.25%) of genes exhibited significant differences in relative abundance comparing the BGISEQ-500 and HiSeq platforms, with a bias towards genes with higher GC content being enriched on the HiSeq platforms.
Conclusion: Our study provides the first set of performance metrics for human gut metagenomic sequencing data using BGISEQ-500. The high accuracy and technical reproducibility confirm the applicability of the new platform for metagenomic studies, though caution is still warranted when combining metagenomic data from different platforms.

**Corresponding Author:**	Junhua Li, Ph.D.<br>BGI<br>shenzhen, guangdong CHINA
**Corresponding Author Secondary Information:**	
**Corresponding Author's Institution:**	BGI

Powered by Editorial Manager® and ProduXion Manager® from Aries Systems Corporation

**Corresponding Author's Secondary Institution:**	
**First Author:**	Chao Fang
**First Author Secondary Information:**	
**Order of Authors:**	Chao Fang
	Huanzi Zhong
	Yuxiang Lin
	Bin Chen
	Mo Han
	Huahui Ren
	Haorong Lu
	Jacob Mayne Luber
	Min Xia
	Wangsheng Li
	Shayna Stein
	Xun Xu
	Wenwei Zhang
	Radoje Drmanac
	Jian Wang
	Huanming Yang
	Lennart Hammarström
	Aleksandar David Kostic
	Karsten Kristiansen
	Junhua Li
**Order of Authors Secondary Information:**	
**Response to Reviewers:**	Response:<br>We thank the Reviewer for the positive comments on the revised version. We have revised the discussion section according to the suggestion made by the reviewer.<br><br>Several studies have demonstrated that accurate identification and quantification of taxa and functions in metagenomics studies could be affected by both the complexity of environmental samples and bioinformatic tools.<br><br>For the most well studied microbiomes, such as the human gut microbiome, typical lengths of 100 bp using the Illumina HiSeq technology, have been extensively applied and the performance has been well demonstrated by numerous high-powered studies, including both reference gene catalog-based metagenome-wide association studies and Metagenomic Phylogenetic Analysis (MetaPhlAn)-based large-scale metagenomics shotgun studies.<br><br>On the other hand, for complex samples form environments such as soil, in which colonizing bacteria remain largely unknow, no reference gene catalogs have been established due to the inherent difficulty of assembly. In these cases, microbial genes were identified using read-based homology searches, and thus, longer read lengths generated from the HiSeq platform would enable more accurate gene identification.
**Additional Information:**	
**Question**	**Response**

Powered by Editorial Manager® and ProduXion Manager® from Aries Systems Corporation

Are you submitting this manuscript to a special series or article collection?	No
**Experimental design and statistics**<br><br>Full details of the experimental design and statistical methods used should be given in the Methods section, as detailed in our Minimum Standards Reporting Checklist. Information essential to interpreting the data presented should be made available in the figure legends.<br><br>Have you included all the information requested in your manuscript?	Yes
**Resources**<br><br>A description of all resources used, including antibodies, cell lines, animals and software tools, with enough information to allow them to be uniquely identified, should be included in the Methods section. Authors are strongly encouraged to cite Research Resource Identifiers (RRIDs) for antibodies, model organisms and tools, where possible.<br><br>Have you included the information requested as detailed in our Minimum Standards Reporting Checklist?	Yes
**Availability of data and materials**<br><br>All datasets and code on which the conclusions of the paper rely must be either included in your submission or deposited in publicly available repositories (where available and ethically appropriate), referencing such data using a unique identifier in the references and in the "Availability of Data and Materials" section of your manuscript.<br><br>Have you have met the above requirement as detailed in our Minimum Standards Reporting Checklist?	Yes

Manuscript

Click here to download Manuscript 2017-12-10 Manuscript-GigaScience-BGISEQ-500-V3.docx

Assessment of the cPAS-based BGISEQ-500 platform for metagenomic sequencing

Abstract

Background: More extensive use of metagenomic shotgun sequencing in microbiome research relies on the development of high-throughput, cost-effective sequencing. Here we present a comprehensive evaluation of the performance of the new high-throughput sequencing platform BGISEQ-500 for metagenomic shotgun sequencing and compare its performance with that of two Illumina platforms.

Findings: Using fecal samples from 20 healthy individuals we evaluated the intra-platform reproducibility for metagenomic sequencing on the BGISEQ-500 platform in a setup comprising 8 library replicates and 8 sequencing replicates. Cross-platform consistency, was evaluated by comparing 20 pairwise replicates on the BGISEQ-500 platform versus the Illumina HiSeq 2000 platform and the Illumina HiSeq 4000 platform. In addition, we compared the performance of the two Illumina platforms against each other.

By a newly developed overall accuracy quality control method, an average of 82.45 million high quality reads (96.06% of raw reads) per sample with 90.56% of bases scoring Q30 and above was obtained using the BGISEQ-500 platform. Quantitative analyses revealed extremely high reproducibility between BGISEQ-500 intra-platform replicates. Cross-platform replicates differed slightly more than intra-platform replicates, yet a high consistency was observed. Only a low percentage (2.02% -3.25%) of genes exhibited significant differences in relative abundance comparing the BGISEQ-500 and HiSeq platforms, with a bias towards genes with higher GC content being enriched on the HiSeq platforms.

Conclusion: Our study provides the first set of performance metrics for human gut metagenomic sequencing data using BGISEQ-500. The high accuracy and technical reproducibility confirm the applicability of the new platform for metagenomic studies, though caution is still warranted when combining metagenomic data from different platforms.

Keywords: BGISEQ-500, Quantitative metagenomic analyses, Next generation sequencing

Data description

To evaluate the performance of the BGISEQ-500 platform for metagenomic sequencing, stool samples were collected from 20 healthy adults in the Stockholm regional area. Fecal DNA was extracted and sequenced on the BGISEQ-500 sequencer. The quality of raw data was evaluated and filtered by an in-house developed quality control (QC) pipeline to obtain high-quality data (see methods and Additional file 2-3 for details). Qualitative and quantitative analyses were conducted to evaluate the intra-platform reproducibility. In addition, data obtained by sequencing of the same fecal DNA samples on the HiSeq 2000 platform and the HiSeq 4000 platform were included for cross-platform comparison (see Fig. 1 and methods for details).

Method

Healthy subject enrollment and sampling

Twenty Swedish healthy adults living in the Stockholm regional area were enrolled as part of a large study cohort: “Characterization of the intestinal microbiome in patients with IgA deficiency”. The detailed inclusion and exclusion criteria were as follows: 1) No diagnosed gastrointestinal problems (inflammatory bowel disease, celiac disease or lactose intolerance); 2) No antibiotic treatment for at least 60 days; 3) No intake of yoghurt products for at least five days prior to sampling. Feces specimens were collected at home by each participant, immediately frozen in the home freezer and transferred to the laboratory on dry ice and kept frozen at -80°C until processed.

DNA extraction

The stool DNA was extracted in accordance with the MetaHIT protocol as described previously [1]. The DNA concentration was estimated by Qubit (Invitrogen).

Library preparation and sequencing

For sequencing using the BGISEQ-500 platform, 500 ng of input DNA were used for library formation and fragmented ultrasonically with Covaris E220 (Covaris, Brighton, UK), yielding 300 to 700 bp fragments. Sheared DNA without size selection was purified with an Axygen™ AxyPrep™ Mag PCR Clean-Up Kit. An equal volume of beads was added to each sample, and DNA was eluted with 45 µl TE buffer. We performed end-repairing and A-tailing with a 2:2:1 mixture of T4 DNA polymerase (ENZYMATICS™ P708-1500), T4 polynucleotide kinase (ENZYMATICS™ Y904-1500) and rTaq DNA polymerase (TAKARA™ R500Z). Twenty ng of purified DNA were used, and enzymes were heat inactivated at 75°C. Adaptors with specific barcodes (Ad153 2B) were ligated to the DNA fragment by T4 DNA ligase (ENZYMATICS™ L603-HC-1500) at 23°C. After the ligation, PCR amplification was carried out. Fifty-five ng of purified PCR products were denatured at 95°C and ligated by T4 DNA ligase (ENZYMATICS™ L603-HC-1500) at 37°C to generate single-strand circular DNA library. Eight barcoded libraries were pooled in equal amounts to make DNA Nanoballs (DNB). Each DNB was loaded into one lane for sequencing.

Sequencing was performed according to the BGISEQ-500 protocol (SOP AO) employing the SE100 mode as described previously [2]. For reproducibility analyses, DNA from the same 8 subjects (S01-S08) were processed twice following the same protocol as described above to serve as library replicates, and one of the DNBs from the same 8 subjects was sequenced twice as sequencing replicates. As shown in Fig. 1, a total of 36 datasets were generated using the BGISEQ-500 platform.

For sequencing using the HiSeq 2000 and 4000 platforms, 1µg DNA was sheared to 350 bp using the Covaris LE220 (Covaris, Inc., Woburn, MA, USA), size selected using AMPure XP beads (Beckman Coulter, Brea, CA, USA). Adapters were then ligated. Twenty libraries were prepared following BGI's protocol [3]. Five libraries were pooled for each lane and sequencing was performed on an Illumina HiSeq

2000 using V3 reagents for 100bp paired-end reads. The base-calling was performed using Illumina pipeline Real Time Analysis (RTA) (version 1.13.48) to process the raw fluorescent images and call sequences.

For both platforms, raw data containing multiple subjects were first split into separate files based on subject specific barcodes. The samples of the 20 subjects sequenced by both BGISEQ-500 and HiSeq 2000 were used to assess the compatibility of metagenomic data across these platforms. For comparison, only the forward reads from HiSeq 2000 were used.

Quality control (QC) of sequencing data

To evaluate the data quality from the two different sequencing platforms, raw FASTQ reads from BGISEQ-500 and HiSeq 2000 were converted into Sanger Phred+33 quality score format and Phred+64 quality score format, respectively [4]. Quality assessment by base position revealed lower quality scores in the beginning of raw reads from the HiSeq 2000 platform compared with BGISEQ-500 and a gradually decreasing trend of quality towards the 3'-end of reads on both platforms (Additional file 1). Considering that a routinely tail trimming QC pipeline would not be sensitive to detection and filtering of reads with randomly distributed low-quality bases, we developed an overall accuracy (OA) control strategy for quality adjustment (Additional file 2). By using this approach, 96.06% of the raw reads remained as high-quality reads which attained an average length of 85 bp and 90.56% of bases scoring Q30 and above. The parameters of sequencing performance both before and after the QC process are presented in Additional file 3.

Alignment and quantification of metagenome content

The high-quality reads of the BGISEQ and HiSeq platforms were then aligned to hg19 using SOAP2.22 (identity ≥ 0.9) to remove human reads (SOAPaligner/soap2 , RRID:SCR_005503) [5]. The retained clean

reads were aligned to the integrated gene catalog (IGC) by using SOAP2.22 (identity ≥ 0.95) [5] . As shown in Additional file 4, the clean reads from BGISEQ-500 reached an average IGC mapping rate of 77.77% and an average unique mapping rate of 63.27%, which is comparable to the mapping rates of reads from the HiSeq 2000 platform. The IGC mapping ratio of subject S01 (54.58%) was significantly lower in the HiSeq 2000 dataset than in the BGISEQ-500 dataset (Additional file 4). Therefore, we eliminated subject S01 for subsequent analysis. To eliminate the influence of different number of reads per sample in intra or cross-platform analyses, uniquely mapped reads were downsized to 20 million for each subject. Gene relative abundance (RA) was calculated based on the down-sized mapped reads as previously described [1]. Relative species abundance in each sample was assessed using MetaPhlAn2 [6].

Intra-platform reproducibility

To estimate the probability distribution of gene occurrence in duplicate experiments, we assessed the expected read count fluctuations based on 20 million IGC uniquely mapped reads (See details in Supplementary method). As shown in Fig. 2a, more than 99.5% genes in replicate 1 (F0) exhibited the expected read count fluctuations in the corresponding sequence replicate 2 (F1) and library replicate 2 (I0) (99% confidence interval, CI). This indicates a high reproducibility of gut microbial gene detection using the BGISEQ-500 platform.

To assess the consistency of relative abundance identification of gut microbial genes, we performed Spearman correlation analysis based on highly-reproducible (HR) genes and species profiles (See details in Supplementary method). Both sequence replicates and library replicates showed high consistency at the gene level (Spearman's rho > 0.91) and species level (Spearman's rho > 0.97) **(**Fig. 2b**).** We further quantified the mean difference between replicates by using area left of the cumulative curve (ALC) (See details in Supplementary method) [7]. The cumulative distributions of replicate differences were plotted (Additional file 5a). The mean gene relative abundance differences between sequence replicates ranged

from 1.008 to 1.323-fold change (Additional file 5b). Similarly, the differences between library replicates ranged from 1.011 to 1.340-fold change (Additional file 5b). Together, these results suggest that very little variation was introduced by library preparation and sequencing processes.

Furthermore, 80,453 and 80,184 HR genes detected in at least 6 pairs of replicates in sequence and library replicates were used for statistical tests, respectively (See details in Supplementary method). Paired-tests of gene abundances revealed no significant difference between BGISEQ-500 technical replicates (FDR < 0.05, Benjamini-Hochberg adjustment). Collectively, these findings demonstrate that the BGISEQ-500 platform, across the entire process of library preparation and sequencing of metagenomic DNA samples, provides highly reproducible and well-controllable results.

Cross-platform consistency

Previously, shotgun metagenomic DNA sequence reads have mostly been generated using Illumina platforms, warranting evaluation of data consistency between the BGISEQ-500 and the Illumina platforms. 91.89% of the genes in the BGISEQ-500 datasets showed expected read count fluctuations in HiSeq 2000 (99% CI), which were less than intra-platform replicates (Fig. 3a). Spearman correlation of HR gene and species profile of cross-platform samples reached 0.724 and 0.948 (Fig. 3b). Compared with intra-platform variations, cross-platform comparison showed a slightly greater difference. The differences in relative abundance between cross-platform groups ranged from 1.409 to 2.015-fold change (Additional file 5b).

Among 349,479 HR genes detected in at least 6 pairs of cross-platform replicates, the relative abundance of 11,350 (3.25%) genes differed significantly between these two platforms (FDR< 0.05, Benjamini-Hochberg adjustment). Among them, 2,051 were detected by paired t-tests, and 9,299 were detected by paired sign tests (See details in Supplementary method). Additionally, these 11,350 genes showed a bimodal distribution in GC-content (Fig. 4a). AT-rich genes were enriched in the BGISEQ-500

dataset. Conversely, the relative abundances of GC-rich genes were higher in the HiSeq 2000 dataset (Fig. 4b). In accordance with the taxonomic annotation of IGC, 25.37% of the genes that differed in relative abundance were assigned to known species (Additional file 6).

Assuming that the abundance of most genes from a species should be even and independent of their GC-content, we conducted robust linear regression analysis of the correlation between the abundance of genes and their GC content for each species (See details in Supplementary method). Based on the genes in the top 20 species exhibiting the most significant differences in abundance, the median of regression coefficient of the BGISEQ-500 dataset was close to 0, namely -0.095 (Fig. 4c, Additional file 7), whereas, the regression coefficient of the HiSeq 2000 dataset was 0.925, indicating a slightly positive correlation between gene abundance and their GC-content. The regression coefficient between all tested genes from the 20 species and their GC contents exhibited a similar tendency (Fig. 4d, Additional file 7). Additionally, generalized linear model (GLM) regression analysis was conducted to investigate the associations between approximate relative species abundance and GC content across the two platforms. MetaPhlAn2 [6] was utilized to generate estimates of relative abundance for each species in each sample. The GC content of each species was retrieved from NCBI. Samples were classified as either high/low abundance (above/below median = 0.2844), either high/low GC content (above/below median = 43.8%) with respect to sequencing platform (BGISEQ-500 or Illumina) (Fig. 5). A log-linear model was used to model the total number of species in each of the 8 categories (abundance high/low, GC content high/low, BGI/Illumina), and a likelihood ratio test then suggested that the association between relative species abundances and their GC content did not vary across the BGISEQ-500 and HiSeq 2000sequencing platforms ($p=0.323$, Chi-squared test) (See details in Supplementary method).

To further document the quantitative consistency and performance regarding GC content observed for the BGISEQ-500 and HiSeq 2000 platforms, the same 20 DNA samples were processed to construct libraries and sequenced on an Illumina HiSeq 4000 platform using the HiSeq 3000/4000SBSKit (300cycles) for

100bp paired-end reads.

The raw sequencing reads were filtered as described above. After QC and removing host reads, an average of 26.35 million clean reads was generated for each sample. The HiSeq 4000 dataset showed comparable high quality and IGC mapping ratio close to that of BGISEQ-500 and HiSeq2000 datasets (Additional file 3). Because of the low number of sequencing reads from the HiSeq 4000 platform, all IGC uniquely mapped reads for each subject (ranging from 13.18 to 21.54 million) were used for validation analyses without downsizing. The forward reads of 19 subjects (subject S01 removed) from HiSeq 4000 were used for further analyses to be consistent with the cross-platform comparison described above.

Interestingly, the Spearman correlation coefficients of HR genes and species profiles between BGISEQ-500 and HiSeq 4000 samples were 0.859 and 0.965, respectively (Fig. 3c). These correlation coefficients were higher than those observed comparing the BGISEQ-500 and HiSeq 2000 datasets (Fig. 3b) and comparing the HiSeq 2000 and HiSeq 4000 datasets (Additional file 8). Statistical analysis revealed smaller quantitative differences between the BGISEQ-500 and HiSeq 4000 platforms than that between the BGISEQ-500 and HiSeq 2000 platforms, with only 6,323 (2.02% of 313,020) HR genes showing significant different relative abundances (FDR< 0.05, Benjamini-Hochberg adjustment). The same bimodal distribution pattern in GC-content were observed among these 6,323 HR genes, with an enrichment of GC-rich genes in the HiSeq 4000 dataset, as well as a slight enrichment of AT-rich genes in BGISEQ-500 dataset. Additionally, the abundance fold changes of these genes were smaller than those observed comparing the BGISEQ-500 and HiSeq 2000 datasets (Fig. 4e, f).

In summary, despite that the HiSeq 2000 platform showed a slight enrichment of reads on a relatively small number of high-GC content genes, metagenomic datasets from BGISEQ-500 and HiSeq 2000 exhibited comparable cross-platform consistency regarding gene detection and quantification. The high cross-platform quantitative consistency was further documented using a HiSeq 4000 dataset, with lower number genes exhibiting differences in abundance between the platforms, and exhibiting the same bimodal

distribution pattern of GC content of these genes.

Discussion

The BGISEQ-500 platform has lately proven its robust performance in connection with several sequencing applications including whole genome sequencing, RNA-seq and small RNA seq [2,8–10]. Unlike these applications, which focus on a single organism, metagenomics deals with a complex mixture of DNA from multiple organisms. One of the key challenges for metagenomics studies is the accurate identification and quantification of taxa and functions in metagenomic samples, which could be affected by both the complexity of environmental samples and the bioinformatic tools.

Though rapid updates of Illumina sequencing techniques have provided up to 300bp long and highly accurate reads, the read lengths available for the BGISEQ-500 were limited to single-end 50bp, single-end 100 bp, and paired-end 2 x 50bp when this study was launched. Hence, we chose the longer mode of single-end 100 bp sequencing for the BGISEQ-500 platform and evaluated its performance.

For the most well studied microbiomes, such as the human gut microbiome, read lengths of 100 bp using the Illumina HiSeq technology, have been extensively applied, and the performance has been well demonstrated by numerous high-powered studies, including both reference gene catalog-based metagenome-wide association studies and Metagenomic Phylogenetic Analysis (MetaPhlAn)-based large-scale quantitative studies [11][12]. On the other hand, for complex samples from environments such as soil, in which colonizing bacteria remain largely unknow, no reference gene catalogs have been established due to the inherent difficulty of assembly. In these cases, microbial genes were thereby identified using read-based homology searches, and thus, longer read lengths would enable more accurate gene identification.

In this work, we have evaluated the performance and validated the performance and robustness of the BGISEQ-500 platform for reference gene catalog-based human gut metagenome studies. We have developed an overall accuracy control-based QC method, which can detect random quality drop within

reads and provide high quality reads with minimal compromise of length. As the most widely used and acknowledged platform in the metagenomics field, datasets generated from Illumina platforms (HiSeq 2000/ 4000) were used throughout the cross-platform comparison.

By comparing metagenomic sequencing datasets from the BGISEQ-500 platform, we demonstrated excellent stability and reproducibility in intra technical replications providing evidence for the robustness and applicability of this new sequencing platform for metagenomics studies. We further demonstrated high consistency between the BGISEQ-500 and the HiSeq 2000 platforms, with only a very small fraction of high GC content genes showing a slight enrichment using the HiSeq 2000 platform. We furthermore compared the datasets from the HiSeq 4000 platform and BGISEQ-500 platform, and corroborated the high cross-platform consistency and platform-dependent GC distribution patterns.

As reported previously [13,14], DNA extraction and library preparation methodology may affect both qualitative analysis and quantitative results in human microbiome research. In addition to that, even though we observed only minor differences in relative gene abundances comparing the BGISEQ-500 and the Illumina platform using single-end 100bp read length, our results clearly point to the importance of using the same platform and technology for metagenomics studies in order to avoid the possible introduction of platform-dependent differences. In cases were comparison of data generated on different platform is desirable, the possible platform-dependent confounding effects should be evaluated by well-designed analyses detecting possible confounding factors and biases before conclusions are drawn.

Finally, the results described in this paper emphasize the need for future use of benchmarking controls including sequencing of defined microbial communities to elucidate the nature of possible biases associated with different preparation methodologies and sequencing platforms.

Declarations

List of abbreviations

cPAS, combinatorial Probe-Anchor Synthesis;

DNB, DNA Nanoball;

QC, quality control;

OA, overall accuracy;

IGC, integrated gene catalog;

RA, relative abundance;

ALC, Area Left of the cumulative Curve;

HR, highly-reproducible;

GLM, generalized linear model

Ethics approval and consent to participate

This study was approved by the Institutional Review Board of Karolinska University Hospital (2016/2502-31/2) and the radiation protection committee of the Karolinska hospital (K2016-4511).

Consent for publication

Not applicable

Competing interests

The authors declare that Chao Fang, Huanzi Zhong, Yuxiang Lin, Bin Chen, Mo Han, Huahui Ren, Haorong Lu, Min Xia, Wangsheng Li, Xun Xu, Wenwei Zhang, Radoje Drmanac, Jian Wang, Huanming Yang, Karsten Kristiansen and Junhua Li are employees of BGI.

Funding

This study was supported by the National Key Research and Development Program of China (No. 2017YFC0909700), the National Natural Science Foundation of China (No.31601073), the Shenzhen Municipal Government of China (No.JSGG20160229172752028), the Shenzhen Municipal Government of China Peacock Plan (No. KQTD20150330171505310) , the American Diabetes Association Pathway Award #1-17-INI-13 (A.D.K.), Smith Family Foundation Award (A.D.K.), NIH/ NHGRI T32 HG002295, PI: Park, Peter J (J.M.L.) and NIH/ NIGMS T32 GM074897, PI: Lin, Xihong & Huttenhower, Curtis (S.S.).

Authors' contributions

J.L. and K.K. conceived and directed the project. J.L. routinely managed the project at BGI-Shenzhen. L.H. was responsible for collection of fecal samples. M.H, W.Z and R.D contributed to metagenomic library construction for the BGISEQ-500 platform. H.Z., B.C., H.L., M.X., and W.L. designed the technical replicates and sequencing experiments. C.F. developed the quality control method for BGISEQ-500 sequencing data. J.L., H.Z. and C.F., designed the analyses. C.F., H.Z., H.R. and Y.L. performed the bioinformatic analyses. H.R contributed to the statistics methods. J.M.L. and S.S. conducted the MetaPhlAn2 results and GLM analysis. H.Z., C.F., J.L., K.K., J.M.L. and A.D.K. interpreted the data. L.H., X.X., J.W. and H.Y. participated in text revision and discussions. C.F., H.Z., and J.M.L. wrote the first version of the manuscript. J.L., K.K. and A.D.K. revised the manuscript.

Acknowledgements

We thank all the volunteers participating in this study. We thank Dr. Yin Shan for providing useful statistical suggestions. We thank Dr. Hui Jiang, Yanyan Zhang and Xia Zhao for their useful suggestions and discussion on metagenomic library construction. We thank Dr. Huijue Jia and Dr. Lise Madsen for

helpful discussion on results. We also thank Dr. Zhe Zhang for helpful discussion and suggestions on the revised manuscript. We gratefully acknowledge colleagues at BGI for DNA extraction, library preparation and sequencing experiments and helpful discussions.

Availability of supporting data

Metagenomic sequencing data for all samples have been deposited in the European Bioinformatics Institute (EBI) database under accession code PRJEB35961. Supporting data is also available from the *GigaScience* GigaDB repository[14].

Tables and Figures

Figure 1 Schematic model summarizing the study design and analysis strategy

Schematic diagram depicting the process of data generation, including collection of fecal samples and extraction of DNA from 20 healthy subjects, library preparation, and sequencing strategy for BGISEQ-500 and HiSeq 2000. Each circle indicates one independent subject, with subject ID shown in the circle. For BGISEQ-500, each sample was sheared and tagged with a unique barcode to prepare libraries, then equal amounts of DNA fragments from 8 samples were pooled together for DNB formation, loading, and sequencing. In total, 20 samples were sequenced in 3 lanes (F0, G0 and H0). Of them, DNA from 8 subjects (S01-S08) were utilized to perform library construction and sequencing twice; the corresponding 8 paired datasets from lane I0 (green) and lane F0 (blue) were considered as library replicates. DNBs from the same 8 subjects were loaded and sequenced twice to generated 8 paired sequencing replicates (lane F0 and lane F1). Twenty datasets from HiSeq 2000 were also generated in this study. The detailed assessment and comparison analyses of metagenomic datasets between intra- and inter-platforms are shown below.

Figure 2 Evaluation of intra platform reproducibility

(a) Detecting mapped read count fluctuations of genes between intra-platform replicates. Unique IGC mapped reads were downsized to 20 million for each subject and the read count fluctuations were estimated (See details in Supplementary method). The x axis represents mapped read counts of a gene in replicate 1(F0), and the y axis represents mapped read counts of that gene in replicate 2 (F1 as sequencing replicate and I0 as library replicate). The area bordered by the red line represents the 99% confidence interval (CI) of genes showing the expected read count fluctuations in their replicates. The dashed line indicates that, at 99% CI, genes with greater than or equal to 10 reads in replicate 1 (x axis) could be detected (with mapped reads great than or equal to 1) in replicate 2 (y axis).

(b) Spearman’s correlation coefficient. Genes with greater than or equal to 10 mapped reads per sample

were retained as highly-reproducible genes and used for Spearman correlation analysis. Both library and sequence replicates showed very high correlations at the gene levels (0.930 and 0.926) and species levels (0.984 and 0.989).

Figure 3 Evaluation of inter-platform consistency

For 19 cross platform replicates at 99% CI, 91.89% genes in the BGISEQ-500 datasets showed the expected mapped read count fluctuations using HiSeq 2000 **(a)**. The Spearman correlation analyses revealed high agreement within 19 pair of platform replicates between BGISEQ-500 and HiSeq 2000 **(b**, an average Spearman's rho of 0.724 at gene level (top) and 0.948 at species level (bottom)) and between BGISEQ-500 and HiSeq 4000 (**c,** an average Spearman's rho of 0.859 at gene level (top) and 0.965 at species level (bottom)).

Figure 4 GC-content distributions of genes that differed significantly in abundance between platforms

Density curves **(a)** showing a comparison of GC-content distributions of the total 9.9 million IGC genes (blue), all 349,479 highly-reproducible (HR) genes (green) and all 11,350 genes that differed significantly in abundance between the two platforms (red line).

2-dimensional plot **(b)** showing the GC-content distribution of genes that differed significantly in abundance between the BGISEQ-500 and HiSeq 2000 platforms. The x axis indicates the GC-content of genes, the y axis indicates fold-changes of gene relative abundance (RA), which is calculated by log10 transformed mean RA in the HiSeq 2000 datasets/ mean RA in the BGISEQ-500 datasets.

Density histograms **(c, d)** showing the coefficients of robust linear model for relative abundance of genes from top 20 species and their GC content for genes that differed significantly in abundance between the two platforms (c) and for all HR genes (d)

Density curves **(e)** and 2-dimensional plot **(f)** showing the GC-content distributions of HR genes that

differed significantly in abundance between the BGISEQ-500 and Hiseq 4000 platforms.

Figure 5 Comparison of relative species abundance between BGISEQ-500 and HiSeq 2000

Averaged microbial abundance calculated with Metaphlan2 across BGI replicates plotted against microbial abundance for the corresponding Illumina replicates for all samples. Species are colored by GC content.

Additional file 1 Base quality assessment

Quality score heatmap showing the distribution of base Phred scores of all raw SE100 reads from BGISEQ-500 (a) and all forward reads from HiSeq 2000 platform.

Additional file 2 Quality control with Overall Accuracy (OA) control strategy

(a) The distribution of per base Phred score (top, orange), per base accuracy (middle, olive) and the overall accuracy (OA) curve of a randomly selected read from the BGISEQ-500 platform (bottom, green) (See details of the OA-based QC pipeline in Supplementary methods)

(b) Identify QC parameters based on overall accuracy and high-quality reads ratio

Box plots showing the average OA of high-quality reads (green) and high-quality reads ratio (grey) based on different $OA_{fragment}$ thresholds. To balance data accuracy and retention rate of high-quality reads after filtering, we chose $OA_{fragment}$ as 0.8. Based on this cutoff, 96.06% of the raw reads were retained as high-quality reads with the average OA value greater than 90%.

Additional file 3 Summary of data production and quality control.

Additional file 4 Assessment of reference coverage for metagenomic sequencing data

After QC and removing potential human-related reads, an average of 95.98% and 97.91% of raw reads were obtained from BGISEQ-500 (blue) and HiSeq 2000 (red) respectively, and were defined as clean reads (left panel). For the BGISEQ-500 platform, an average of 77.77% of total clean reads could be mapped to IGC; the averaged mapping rate on HiSeq 2000 was 75.45% (middle panel). Additionally, HiSeq 2000 dataset from subject S01 showed a low IGC mapping rate of 54.58% and was subsequently marked as an outlier and removed before cross platform comparison. For both platforms, more than 62% of total clean reads were uniquely mapped (right panel) and used for further quantification analysis.

Additional file 5 ALC value for sequencing, library and cross-platform replicates

The ALC value is the area left of the cumulative distribution curve: Thus, a low ALC value denotes high reproducibility. The ALC value is determined by the relative abundance differences of highly-reproducible genes between replicates. The x axis represents log2 transformed difference-folds, and the y axis represents the cumulative proportion of gene relative abundance difference for intra- and cross-platform (a). The box plot shows the estimated fold-change differences in relative gene abundance between replicates calculated by ALC values (b).

Additional file 6 Summary of species annotation for the genes that differed significantly in abundance between the BGISEQ500 and the HiSeq 2000 platform.

Additional file 7 Robust linear regression analysis between gene relative abundance and GC content

Additional file 8 Spearman's correlation between datasets from HiSeq 2000 and HiSeq 4000

Reference

1. Qin J, Li Y, Cai Z, Li S, Zhu J, Zhang F, et al. A metagenome-wide association study of gut microbiota in type 2 diabetes. Nature. Nature Publishing Group; 2012;490:55–60.

2. Huang J, Liang X, Xuan Y, Geng C, Li Y, Lu H, Qu S, Mei X, Chen H, Yu T, Sun N, Rao J, Wang J, Zhang W, Chen Y, Liao S, Jiang H, Liu X, Yang Z, Mu F, Gao S. A reference human genome dataset of the BGISEQ-500 sequencer. Gigascience. 2017 May 1;6(5):1-9. doi: 10.1093/gigascience/gix024.

3. Taylor P, Liu L, Hu N, Wang B, Chen M, Wang J, et al. A brief utilization report on the Illumina HiSeq 2000 sequencer. Mycology. 2011;2:169–91.

4. Ewing B, Green P. Base-calling of automated sequencer traces using phred. II. Error probabilities. Genome Res. 1998;8:186–94.

5. Li J, Jia H, Cai X, Zhong H, Feng Q, Sunagawa S, et al. An integrated catalog of reference genes in the human gut microbiome. Nat. Biotechnol. 2014;32:834–41.

6. Truong DT, Franzosa EA, Tickle TL, Scholz M, Weingart G, Pasolli E, et al. MetaPhlAn2 for enhanced metagenomic taxonomic profiling. Nat. Methods. 2015;12:902–3.

7. Mestdagh P, Hartmann N, Baeriswyl L, Andreasen D, Bernard N, Chen C, et al. Evaluation of quantitative miRNA expression platforms in the microRNA quality control (miRQC) study. Nat. Methods. 2014;11:809–15.

8. Mak SST, Gopalakrishnan S, Carøe C, Geng C, Liu S, Sinding MS, Kuderna LFK, Zhang W, Fu S, Vieira FG, Germonpré M, Bocherens H, Fedorov S, Petersen B, Sicheritz-Pontén T, Marques-Bonet T, Zhang G, Jiang H, Gilbert MTP. Comparative performance of the BGISEQ-500 vs Illumina HiSeq2500 sequencing platforms for palaeogenomic sequencing. Gigascience. 2017 Aug 1;6(8):1-13. doi: 10.1093/gigascience/gix049.

9. Fehlmann T, Reinheimer S, Geng C, Su X, Drmanac S, Alexeev A, et al. cPAS-based sequencing on the BGISEQ-500 to explore small non-coding RNAs. Clin. Epigenetics. 2016;8:123.

10. Zhang B, Zhang W, Nie RE, Li WZ, Segraves KA, Yang XK, et al. Comparative transcriptome analysis of chemosensory genes in two sister leaf beetles provides insights into chemosensory speciation. Insect Biochem. Mol. Biol. 2016;79:108–18.

11. Liu R, Hong J, Xu X, Feng Q, Zhang D, Gu Y, et al. Gut microbiome and serum metabolome alterations in obesity and after weight-loss intervention. Nat. Med. 2017;23:859–68.

12. Zhernakova A, Kurilshikov A, Bonder MJ, Tigchelaar EF, Schirmer M, Vatanen T, et al. Population-based metagenomics analysis reveals markers for gut microbiome composition and diversity. Science (80-.). 2016;352:565–9.

13. Jones MB, Highlander SK, Anderson EL, Li W, Dayrit M, Klitgord N, et al. Library preparation methodology can influence genomic and functional predictions in human microbiome research. Proc Natl Acad Sci U S A. 2015;112:1519288112-.

14. Hart ML, Meyer A, Johnson PJ, Ericsson AC. Comparative evaluation of DNA extraction methods from feces of multiple host species for downstream next-generation sequencing. PLoS One. 2015;10.

15. Fang, C; Zhong, H; Lin, Y; Chen, B; Han, M; Ren, H; Lu, H; Luber, J, M; Xia, M; Li, W; Stein, S; Xu, X; Zhang, W; Drmanac, R; Wang, J; Yang, H; Hammarström, L; Kostic, A, D; Kristiansen, K; Li, J (2017): Supporting data for "Assessment of

the cPAS-based BGISEQ-500 platform for metagenomic sequencing" GigaScience Database. http://dx.doi.org/10.5524/100395

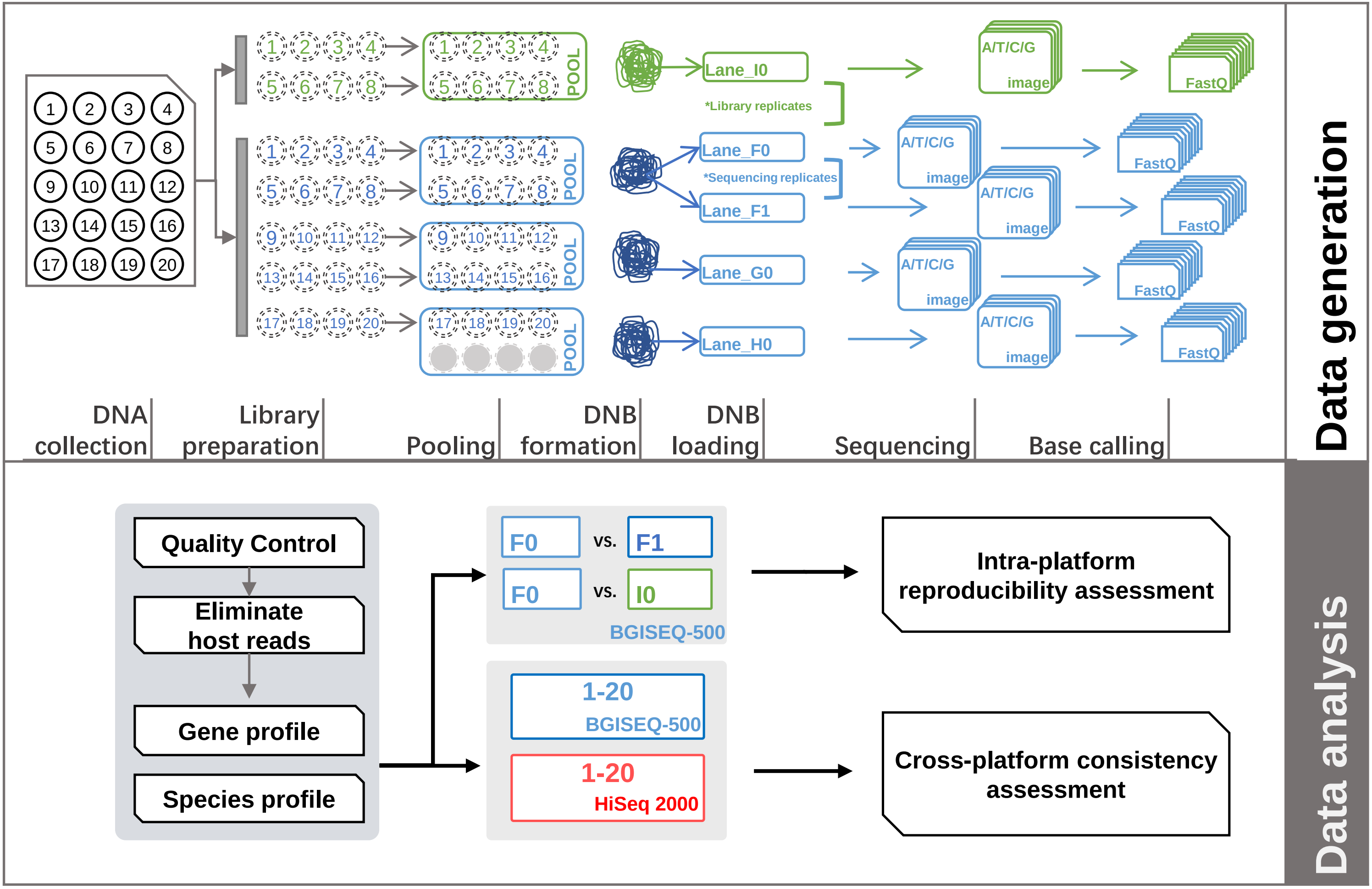

Data generation
DNA collection
Library preparation
Pooling
DNB formation
DNB loading
Sequencing
Base calling
POOL
Lane_I0
*Library replicates
Lane_F0
*Sequencing replicates
Lane_F1
Lane_G0
Lane_H0
A/T/C/G image
FastQ
Data analysis
Quality Control
Eliminate host reads
Gene profile
Species profile
F0 vs. F1
F0 vs. I0
BGISEQ-500
1-20 BGISEQ-500
1-20 HiSeq 2000
Intra-platform reproducibility assessment
Cross-platform consistency assessment

Figure 2

Click here to download Figure Figure 2.pdf

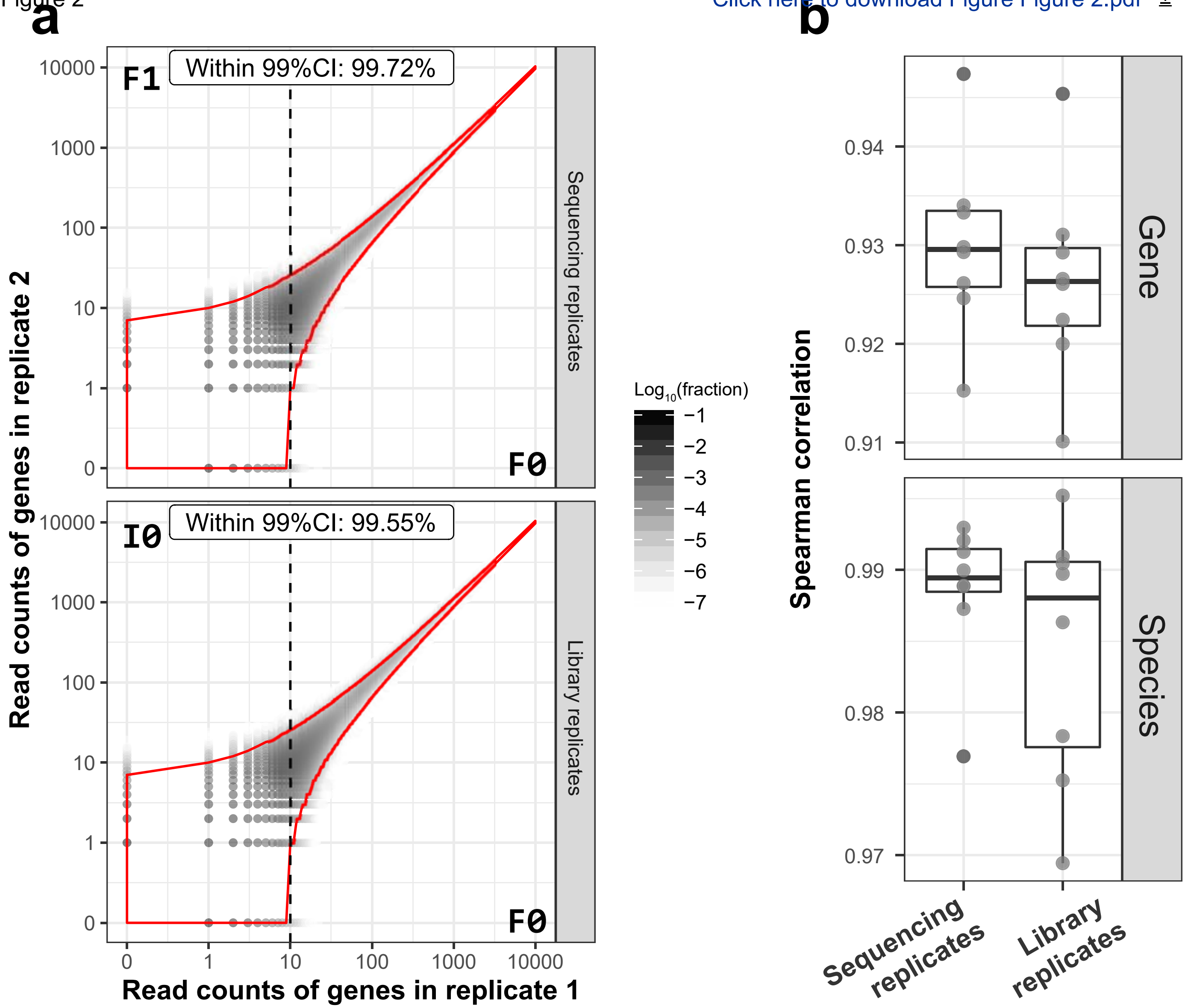

Figure 3

Click here to download Figure Figure 3.pdf

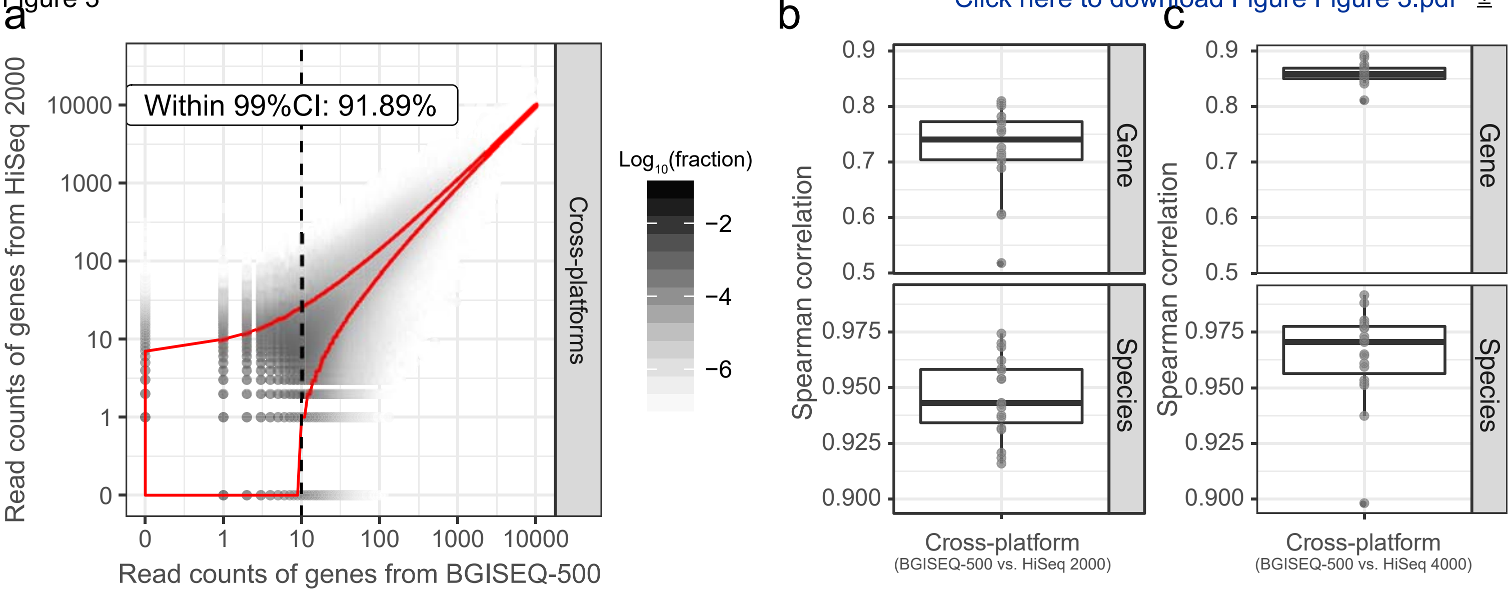

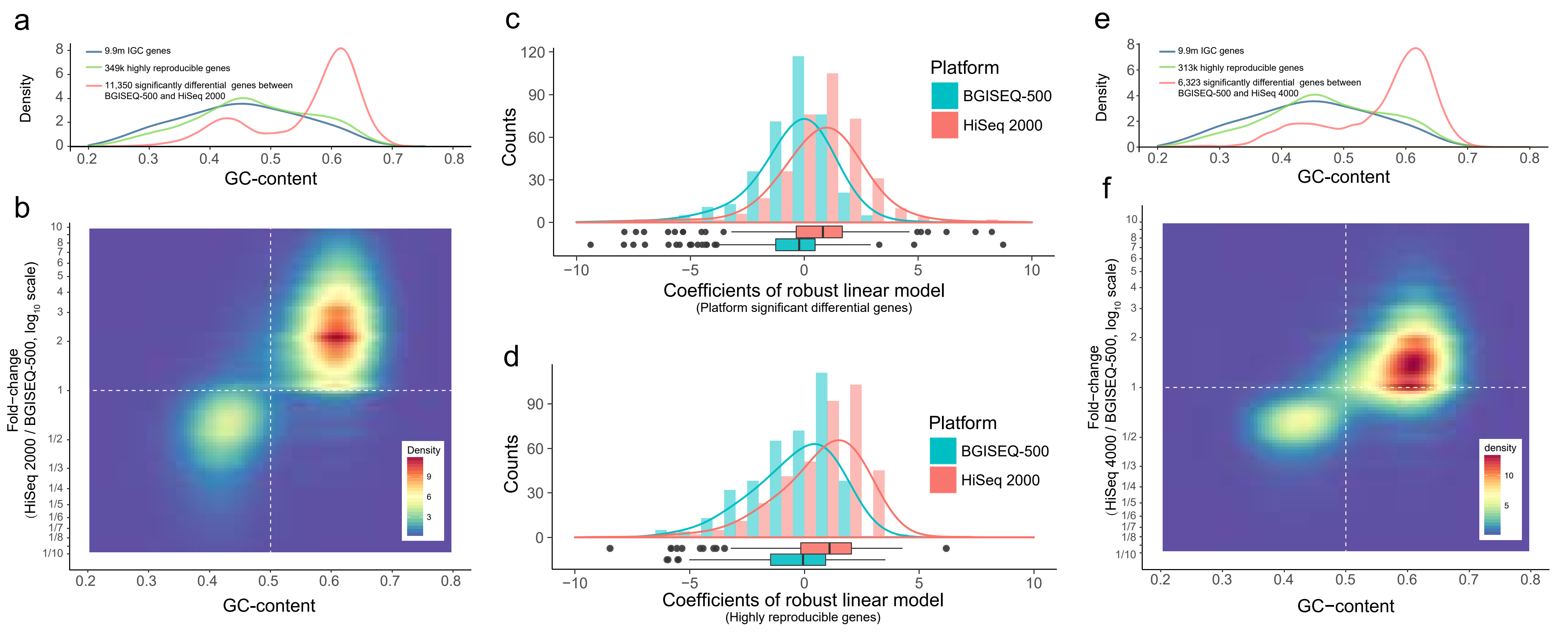
a
Density
9.9m IGC genes
349k highly reproducible genes
11,350 significantly differential genes between BGISEQ-500 and HiSeq 2000
GC-content
b
Fold−change (HiSeq 2000 / BGISEQ-500, log10 scale)
Density
GC-content
c
Counts
Platform
BGISEQ-500
HiSeq 2000
Coefficients of robust linear model
(Platform significant differential genes)
d
Counts
Platform
BGISEQ-500
HiSeq 2000
Coefficients of robust linear model
(Highly reproducible genes)
e
Density
9.9m IGC genes
313k highly reproducible genes
6,323 significantly differential genes between BGISEQ-500 and HiSeq 4000
GC-content
f
Fold−change (HiSeq 4000 / BGISEQ-500, log10 scale)
density
GC−content

Figure 5

Click here to download Figure Figure 5.pdf

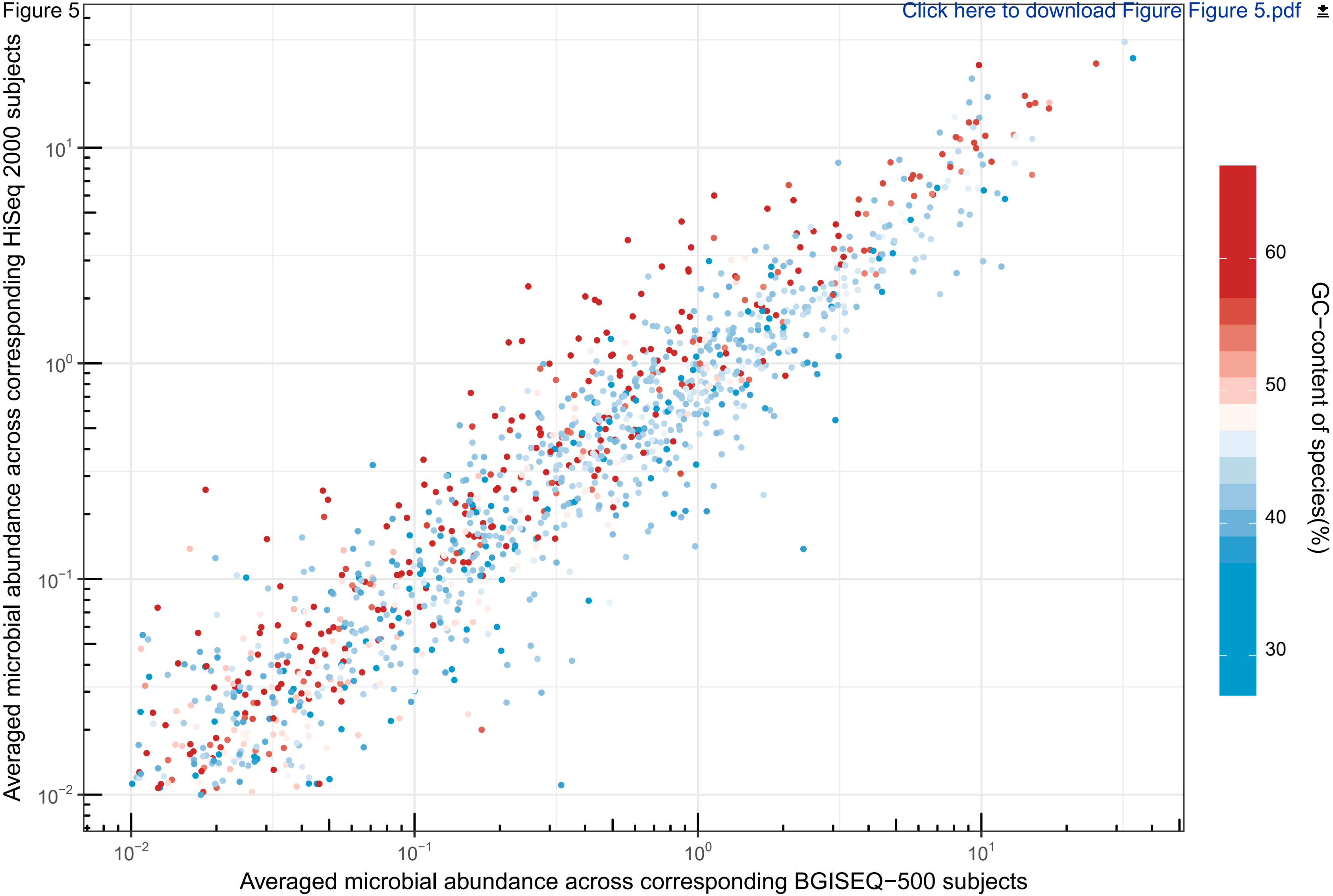

Supplementary Material 1

Click here to access/download
Supplementary Material
Additional file 1.pdf

Supplementary Material 2

Click here to access/download
Supplementary Material
Additional file 2.pdf

Supplementary Material 3

Click here to access/download

Supplementary Material

Additional file 3.xlsx

Supplementary Material 4

Click here to access/download
Supplementary Material
Additional file 4.pdf

Click here to access/download
Supplementary Material
Additional file 5.pdf

Supplementary Material 6

Click here to access/download
Supplementary Material
Additional file 6.xlsx

Click here to access/download
Supplementary Material
Additional file 7.xlsx

Supplementary Material 8

Click here to access/download
Supplementary Material
Additional file 8.pdf

Supplementary Method

Click here to access/download
Supplementary Material
Supplementary Method.doc

Rebuttal Figure 1

Click here to access/download
Supplementary Material
Rebuttal Figure 1.pdf

 Click here to download Personal Cover 2017-12-10 Coverletter-GigaScience-BGISEQ-500-Final.docx

Dear Editor,

On behalf of all the authors, we hereby submit a revised manuscript, originally entitled: "*Assessment of the cPAS-based BGISEQ-500 platform for metagenomic sequencing*".

We have revised the Discussion section to comply with the suggestions of the reviewers as detailed in the Point-by-Point response.

We hope that the manuscript after this revision is acceptable for publication in GigaScience.

Yours sincerely,

Junhua Li, PhD

BGI-Shenzhen

Email: lijunhua@genomics.cn

In order to try the double-blind peer reviewing process, author information has been removed from the text:

Chao Fang†[1,2,3], Huanzi Zhong†[1,2,4], Yuxiang Lin[1,2,3], Bin Chen[1,2,3], Mo Han[1,2,3], Huahui Ren[1,2,3], Haorong Lu[1,2], Jacob M. Luber[5,6,7,8,9,10], Min Xia[1,2], Wangsheng Li[1,2], Shayna Stein[6,11,12], Xun Xu[1,2], Wenwei Zhang[1], Radoje Drmanac[1], Jian Wang[1,13], Huanming Yang[1,13], Lennart Hammarström[14], Aleksandar D. Kostic[7,8,10], Karsten Kristiansen[1,2,4], Junhua Li*[1,15,2,3]

*Corresponding author: Junhua Li Ph. D, lijunhua@genomics.cn

† Equal contributor

1. BGI-Shenzhen, Shenzhen 518083, China.
2. China National GeneBank, BGI-Shenzhen, Shenzhen 518120, China
3. Shenzhen Key Laboratory of Human commensal microorganisms and Health Research, BGI-Shenzhen, Shenzhen 518083, China.
4. Laboratory of Genomics and Molecular Biomedicine, Department of Biology, University of Copenhagen, 2100 Copenhagen Ø, Denmark
5. Program in Bioinformatics and Integrative Genomics, Division of Medical Sciences, Harvard Medical School, Boston, MA 02115, USA
6. Graduate School of Arts and Sciences, Harvard University, Cambridge, MA, 02138, USA
7. Section on Pathophysiology and Molecular Pharmacology, Joslin Diabetes Center, Boston, MA 02215, USA
8. Section on Islet Cell and Regenerative Biology, Joslin Diabetes Center, Boston, MA 02215, USA
9. Department of Biomedical Informatics, Harvard Medical School, Boston, MA 02115, USA
10. Department of Microbiology and Immunobiology, Harvard Medical School, Boston, MA 02115, USA
11. Department of Biostatistics and Computational Biology, Dana Farber Cancer Institute, Boston, MA 02115, USA
12. Department of Biostatistics, Harvard TH Chan School of Public Health, Boston, MA 02215, USA
13. James D. Watson Institute of Genome Sciences, Hangzhou 310058, China
14. Division of Clinical Immunology and Transfusion Medicine, Department of Laboratory Medicine, Karolinska University Hospital, Huddinge, SE-14186 Stockholm, Sweden
15. School of Bioscience and Biotechnology, South China University of Technology, Guangzhou 510006, China
