# Supplementary figures and images for: Assessment of the cPAS-based BGISEQ-500 platform for metagenomic sequencing
Source: Gigascience. 2017 Dec 23;7(3):gix133. doi: 10.1093/gigascience/gix133 (PMC5848809; doi:10.1093/gigascience/gix133)

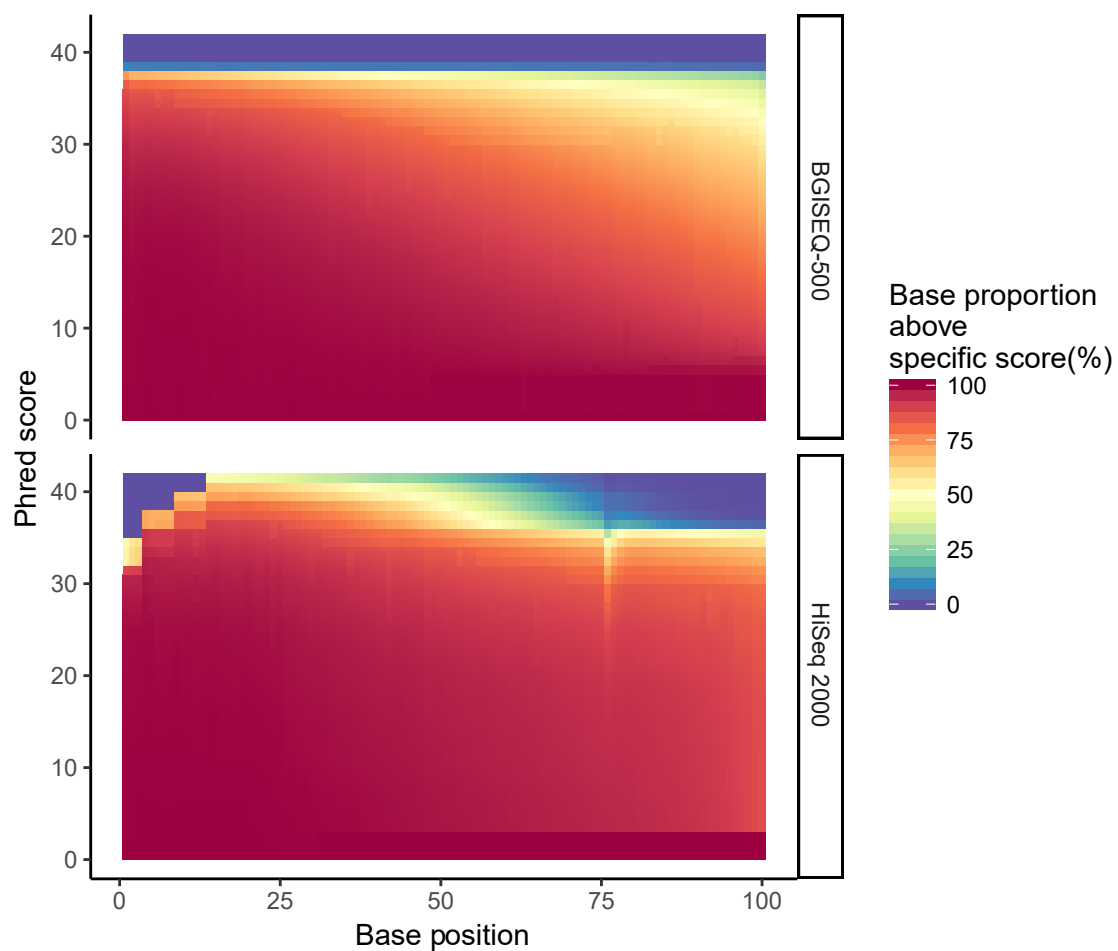

Supplement: Supplemental material [file gix133_supp.zip › Additional file 1.pdf]

a

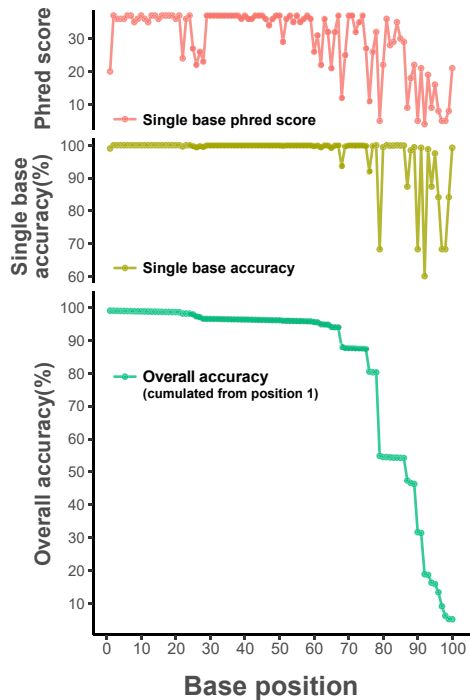

b

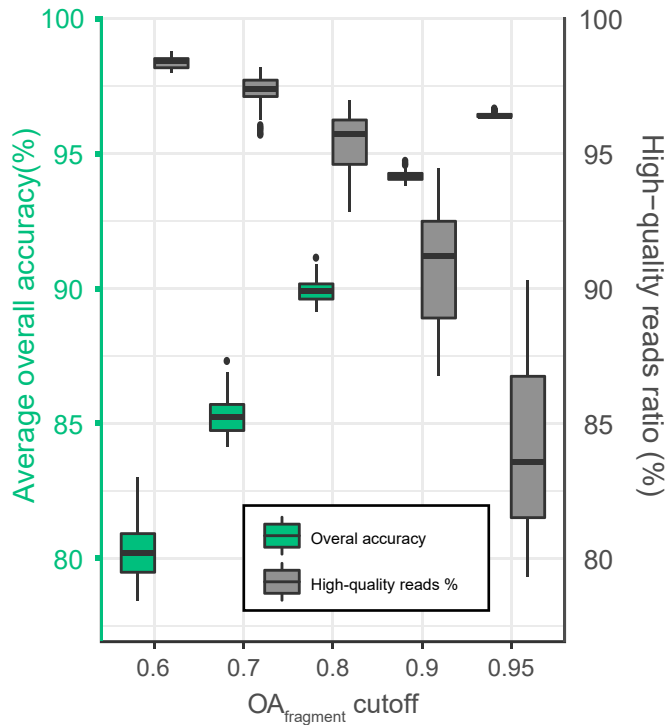

Supplement: Supplemental material [file gix133_supp.zip › Additional file 2.pdf]

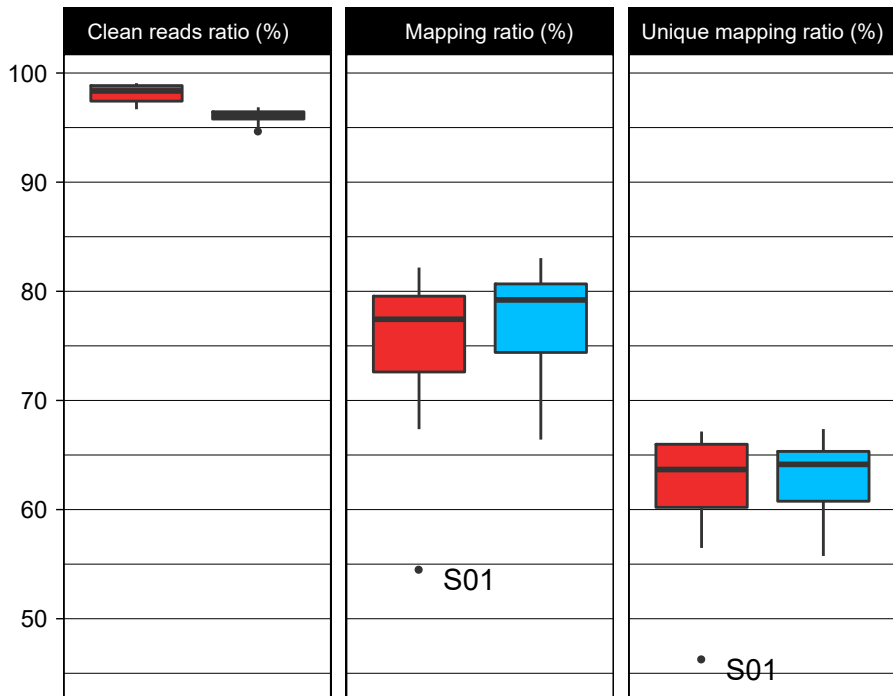

Platform

HiSeq 2000

BGISEQ-500

Supplement: Supplemental material [file gix133_supp.zip › Additional file 4.pdf]

**a**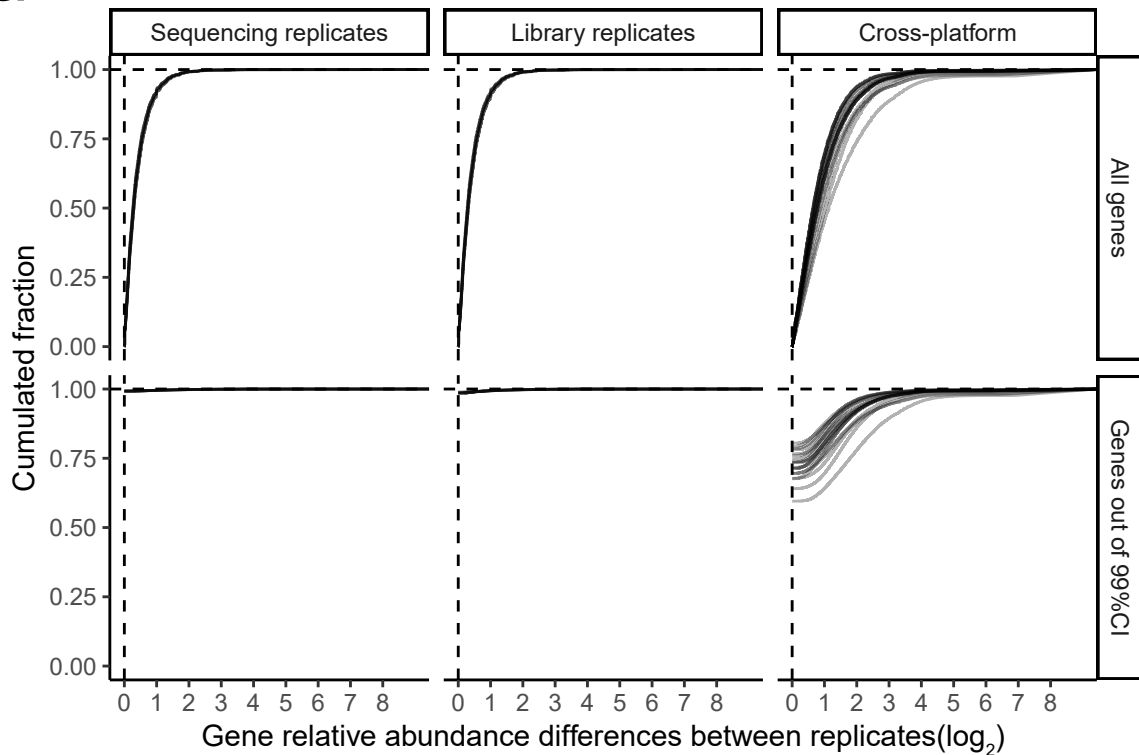**b**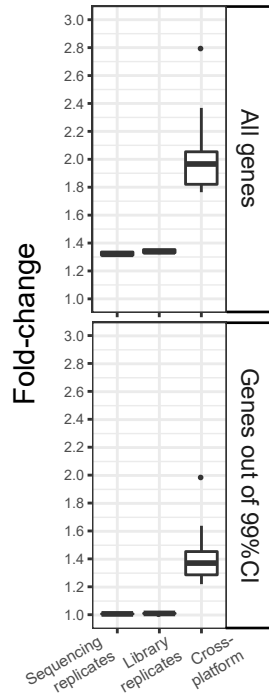

Supplement: Supplemental material [file gix133_supp.zip › Additional file 5.pdf]

Spearman correlation

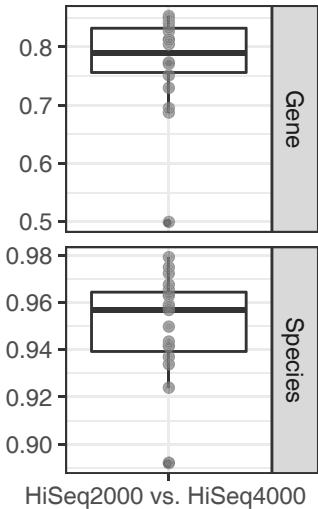

Supplement: Supplemental material [file gix133_supp.zip › Additional file 8.pdf]
