# Supplementary material for: Assessment of the cPAS-based BGISEQ-500 platform for metagenomic sequencing
Source: Gigascience. 2017 Dec 23;7(3):gix133. doi: 10.1093/gigascience/gix133 (PMC5848809; doi:10.1093/gigascience/gix133)
Supplement: Response_to_Reviewer_Comments_Original_Submission.pdf [file gix133_response_to_reviewer_comments_original_submission.pdf]

Author's Response To Reviewer Comments

Reviewer 1:
The paper presents an example of sequencing human gut metagenomics based on BGISEQ-500 platform. Through using a variety of statistical methods to compare intra-platform reproducibility and cross-platform consistency in different metagenomic sequencing datasets, BGISEQ-500 platform has a good performance. Besides, this work is a good benchmark for future metagenomic sequencing with different methodologies and platform. So it presents a topic of interest to the researchers in the related areas.

We thank the Reviewer for the positive comment.

Minor concerns:
1. In section "Library preparation and sequencing ", there is a lack of explanation of different amount of input DNA for sequencing between BGISEQ-500 platform and HiSeq 2000 platform.

We thank the reviewer for this comment.
In this study, we used lower amounts of input DNA for the BGISEQ-500 platform, which reflects the different library construction strategies adopted by two platforms.
For the BGISEQ500 platform we constructed metagenomic libraries of DNA fragments without size selection, whereas the manufacturer's instruction for the HiSeq 2000 suggests a size selection step after shearing of DNA. The latter therefore required a higher amount of input DNA to ensure enough DNA fragments for adaptor ligation.

2.In section "Cross-platform consistency", HR genes detected in at least 6 pairs of cross-platform replicates is more than three times the number of it in sequence and library replicates, which need to be discussed.

We thank the reviewer for this suggestion.
First, several studies have demonstrated the high inter-individual heterogeneity of the human gut microbiome. To control the false positive discovery rate, we only perform statistical test on HR genes detected in least 6 pairs of replicates to avoid a large number of unreliable tests.
Second, the greater number of HR genes used for cross-platform replicates reflected the larger sample size for cross-platform comparisons.
We used only 8 pairs of sequencing and library replicates, and the selected HR genes detected in at least 6 pairs corresponded to an occurrence of 75% (6/8) or greater. By contrast, by analyzing19 pairs of cross-platform replicates, the corresponding occurrence of HR genes detected in at least 6 pairs was much lower (6/19), but ensured more HR genes for comparison.

Reviewer #2:
The authors present a study of the feasibility of metagenomics sequencing using the BGISEQ-500 sequencing platform. For this purpose, a number of new stool samples were sequenced on both the BGISEQ-500 and Illumina HiSeq 2000 sequencing platforms. The authors further developed a new method to quality control the sequencing reads produced by the BGISEQ-500 platform. In general, the authors find the BGISEQ-500 to be highly reproducible and accurate.

Nevertheless, there are several issues with manuscript.

Major issues:
The authors compare against the Illumina HiSeq 2000 sequencing platform even though it is not commercially available anymore. I don't understand why the authors did not compare to an Illumina HiSeq 2500 sequencing platform. One large difference between the HiSeq 2000 and the HiSeq 2500 is the read length, which is known to strongly influence mapping results in metagenomics.

We thank the Reviewer for this very valid comment.
In revised version, we have compared the metagenomic datasets obtained by sequencing the same 20 DNA samples on the HiSeq 4000 platform with datasets obtained by using the BGISEQ-500 and HiSeq 2000 platforms.
This comparison revealed that the mean Spearman's coefficient of HR gene profiles between BGISEQ-500 and HiSeq 4000 replicates reached 0.859, which was higher than the Spearman's coefficient comparing BGISEQ-500 and HiSeq 2000 (Rho=0.724) (Figure 3c). Furthermore, we observed that a lower number of HR genes (6, 323; 2.02%) exhibited significant differences in relative abundances between the BGISEQ-500 platform and the HiSeq 4000 platform compared to the figures obtained by comparing the BGISEQ-500 platform and the HiSeq 2000 platform. We also observed the same bimodal GC distribution of these genes, with GC-rich genes being enriched using the HiSeq 4000 platform and AT-rich genes being enriched using the BGISEQ-500 platform (Figure 4e, f). Taken together, these findings led to the conclusion that BGISEQ-500 showed high consistency with the most widely used sequencing platforms (including HiSeq 2000 and HiSeq 4000) in quantitative metagenomics. We have expanded the text to describe the comparison between the BGISEQ-500 platform and the HiSeq 4000 platform. Thus, the manuscript has now been revised accordingly (Page 7, lines 19-24; Page 8, lines1-16). We hope that this expansion is sufficient to address the platform issues raised by the reviewer.

We also agree with the reviewer’s opinion that read length is a source of bias on its own, partly because it is more difficult to detect homology for short reads [1,2]. Still, metagenomic shotgun sequencing, with typical lengths of 100 bp using the Illumina HiSeq technology, has been extensively applied in recent large-scale gut microbial studies [3,4].
To eliminate the potential impacts of read length, we compared the quantitative metagenomics performance of the two platforms using the same sequencing read length of 100bp. To ensure the alignment accuracy, only reads which mapped uniquely to the reference gene catalogue were retained for further qualitative and quantitative analyses, similar to what has been widely used in several metagenome-wide association studies [4,7,8].
The quantitative performances of paired-end and single-end 100 reads of HiSeq platforms will be discussed in the response to the next question.

2. The authors state: "For comparison, only the forward reads from HiSeq 2000 were used." One of the strengths of Illumina sequencing is the paired end capability. In principle, this should also be available for the BGISEQ-500 platform. The authors ought to use the forward and reverse reads for their comparison.

We thank the Reviewer for the comments.

When we initiated the project, the initial BGISEQ-500 sequencing technology only produced reads of 50bp using single- and paired-end sequencing [5], and only 100bp using single-end (SE) sequencing [6]. Hence, we have chosen the longer and most commonly used read length of SE 100bp from BGISEQ-500 platform. For HiSeq platforms, only the forward reads from PE 100bp reads were used to simulate the SE 100bp mode of BGISEQ-500 platform.
To better address the reviewer's concern, we evaluated the quantitative metagenomics performance using SE100 and PE100 reads from the HiSeq platforms, including the IGC mapping ratios and Spearman's correlation based on gene and species levels.

As shown, the IGC mapping ratios of both SE100 and PE100 reads from HiSeq 2000 and HiSeq 4000 platforms were very close to each other, with fold changes (FC) of IGC mapping ratios using SE100 and PE100 of less than 1.01 (FC=1.00338 for HiSeq 2000, FC=1. 00296 for HiSeq 4000) (Rebuttal Fig. 1a). Further, we calculated the Spearman's correlation coefficients of gene profiles between SE100 and PE100 reads mode for the HiSeq 2000 platform and the HiSeq 4000 platform, respectively. The gene abundance profiles from the two modes also showed extremely high correlations to both the HiSeq 2000 platform and the HiSeq 4000 platform (Rebuttal Fig. 1b, c) (Spearman's Rho > 0.91).
These results together suggest that sequencing modes exhibit negligible effects on the reference-based quantitative metagenomics analyses.

Rebuttal Figure 1 Comparison of quantification performance between SE100 and PE100 reads from the HiSeq platforms

3. In their section "Quality control (QC) of sequencing data", the authors mention a new method to quality control sequencing reads. I cannot find an accurate and thorough description of this method. I would also be good motivate the need for this method better. This seems to be a key part of the manuscript and should be extensively reworked. It would further be good to provide this method as free and open-source a software for readers to use.

We thank the reviewer for the positive comment on this newly developed quality control method. Please find the detailed description in the revised Supplementary method (Page 2-3, lines 8-40) and the uploaded Perl scripts is also freely available from GigaScience (XX) and GitHub (https://github.com/Scelta/OAFilter).

4. The authors ought to explain the challenges in metagenomics sequencing compared to other sequencing application better.

We thank the reviewer for this constructive point.
This new platform has proven its performance in relation to several sequencing applications, such as whole genome sequencing, RNA-seq and small RNA seq. In this study, we specially focused on the performance of BGISEQ-500 platform for quantitative comparative metagenomics. All the previous sequencing applications focused on a single organism, whereas metagenomics deals with a mixture of DNA from multiple organisms with highly variable GC content.
As the reviewer mention, one of the challenges for metagenomics should be the limitation of using short reads based homology searches to identify genes similar to reference genes. We

believe that the future rapid updates of sequencing techniques would soon provide longer and even more accurate reads and improve the accuracy for reads-based metagenomic taxonomic and functional annotation and quantification.
We now have added a few sentences in the Discussion section to clarify the current available sequencing strategies of the BGISEQ-500 platform and challenges associated with the use of short length reads-based quantitative metagenomics (Page 9).

Minor issues:
1. Page 2, line 7: The authors mention that they used the same faecal subjects. It would be good if the authors could specify what exactly this means. Are these samples from the same person? The same piece of stool? Before or after homogenization?
We apologize for the confusion.
We extracted faecal DNA once for each subject and we sequenced the same DNA samples from the same subject on the platforms.
We have revised the sentence as “In addition, data obtained by sequencing of the same fecal DNA samples on the HiSeq 2000 platform…” (Page 2, lines 7-8).

2. Page 5, line 2-4: I don't understand why the authors decided to do the following: "To eliminate the influence of different number of reads per sample in intra or cross-platform analyses, unique mapped reads were downsized to 20 million for each subject." According to Suppl. File 3, all samples have more than 30M sequencing reads. I do not understand the motivation to down sample to 20M reads.

Only the uniquely IGC mapped reads, but not all clean reads, were used for cross-platform comparison.
All samples generated from HiSeq 2000 had more than 30M high quality clean reads, while the uniquely IGC mapped reads of each sample ranged from 20.68M to 33.47M (Additional file 3). Hence, we downsized the uniquely mapped reads to 20M for further analyses.

3. The authors mention that GC content and gene abundance are related on page 6. On page 7 they mention that this relationship does not vary between sequencing platforms. The authors need to clarify.
We apologize for the confusion.
On page 6, we firstly reported the relative abundances of only 3.25% HR genes differed significantly between the BGISQ-500 platform and the HiSeq 2000 platform, with enrichment of GC-rich genes on the HiSeq 2000 platform.
We assume that this small fraction of total HR genes could account for very limited bias of cross-platform GC content distribution. Then, we conducted generalized linear regression analysis between abundance of species and their GC content (on page 7). As expected, we found that the relative abundance of species and their GC content did not vary across the BGISEQ-500 and HiSeq 2000 platforms, emphasizing the high quantitative consistency between the two platforms.
We have modified the sentences (Page 7, lines 15-18) to clarify that we observed no relationship between species relative abundance and their GC content.

Reference:

1. Menzel P, Ng KL, Krogh A. Fast and sensitive taxonomic classification for metagenomics with Kaiju. Nat. Commun. 2016;7:11257.
2. Orellana LH, Rodriguez-R LM, Konstantinidis KT. ROCker: accurate detection and quantification of target genes in short-read metagenomic data sets by modeling sliding-window bitscores. Nucleic Acids Res. 2016;gkw900.
3. Zhernakova A, Kurilshikov A, Bonder MJ, Tigchelaar EF, Schirmer M, Vatanen T, et al. Population-based metagenomics analysis reveals markers for gut microbiome composition and diversity. Science (80-.). 2016;352:565–9.
4. Liu R, Hong J, Xu X, Feng Q, Zhang D, Gu Y, et al. Gut microbiome and serum metabolome alterations in obesity and after weight-loss intervention. Nat. Med. 2017;23:859–68.
5. Huang J, Liang X, Xuan Y, Geng C, Li Y, Lu H, et al. A reference human genome dataset of the BGISEQ-500 sequencer. Gigascience. 2017;1–9.
6. Mak SST, Gopalakrishnan S, Carøe C, Geng C, Liu S, Sinding M-HS, et al. Comparative performance of the BGISEQ-500 versus Illumina HiSeq2500 sequencing platforms for palaeogenomic sequencing. Gigascience. 2017;
7. Qin J, Li Y, Cai Z, Li S, Zhu J, Zhang F, et al. A metagenome-wide association study of gut microbiota in type 2 diabetes. Nature. 2012;490:55–60.
8. Li J, Jia H, Cai X, Zhong H, Feng Q, Sunagawa S, et al. An integrated catalog of reference genes in the human gut microbiome. Nat Biotech. 2014;advance on:834–41.
