# Supplementary material for: Assessment of the cPAS-based BGISEQ-500 platform for metagenomic sequencing
Source: Gigascience. 2017 Dec 23;7(3):gix133. doi: 10.1093/gigascience/gix133 (PMC5848809; doi:10.1093/gigascience/gix133)
Supplement: Response_to_Reviewer_Comments_Revision_1.pdf [file gix133_response_to_reviewer_comments_revision_1.pdf]

Author's Response To Reviewer Comments

Response:
We thank the Reviewer for the positive comments on the revised version. We have revised the discussion section according to the suggestion made by the reviewer.

Several studies have demonstrated that accurate identification and quantification of taxa and functions in metagenomics studies could be affected by both the complexity of environmental samples and bioinformatic tools.

For the most well studied microbiomes, such as the human gut microbiome, typical lengths of 100 bp using the Illumina HiSeq technology, have been extensively applied and the performance has been well demonstrated by numerous high-powered studies, including both reference gene catalog-based metagenome-wide association studies and Metagenomic Phylogenetic Analysis (MetaPhlAn)-based large-scale metagenomics shotgun studies.

On the other hand, for complex samples form environments such as soil, in which colonizing bacteria remain largely unknow, no reference gene catalogs have been established due to the inherent difficulty of assembly. In these cases, microbial genes were identified using read-based homology searches, and thus, longer read lengths generated from the HiSeq platform would enable more accurate gene identification.
