# Supplementary material for: Assessment of the cPAS-based BGISEQ-500 platform for metagenomic sequencing
Source: Gigascience. 2017 Dec 23;7(3):gix133. doi: 10.1093/gigascience/gix133 (PMC5848809; doi:10.1093/gigascience/gix133)
Supplement: Reviewer_2_Report_(Original_Submission) -- Daniel Mende — 01 Nov 2017 Reviewed [file gix133_reviewer_2_report_(original_submission).pdf]

**Reviewer Report**

**Title:** Assessment of the cPAS-based BGISEQ-500 platform for metagenomic sequencing

**Version:** Original Submission **Date:** 01 Nov 2017

**Reviewer name:** Daniel Mende

**Reviewer Comments to Author:**

The authors present a study of the feasibility of metagenomics sequencing using the BGISEQ-500 sequencing platform. For this purpose, a number of new stool samples were sequenced on both the BGISEQ-500 and Illumina HiSeq 2000 sequencing platforms. The authors further developed a new method to quality control the sequencing reads produced by the BGISEQ-500 platform. In general, the authors find the BGISEQ-500 to be highly reproducible and accurate.

Nevertheless, there are several issues with manuscript.

Major issues:
1. The authors compare against the Illumina HiSeq 2000 sequencing platform even though it is not commercially available anymore. I don't understand why the authors did not compare to an Illumina HiSeq 2500 sequencing platform. One large difference between the HiSeq 2000 and the HiSeq 2500 is the read length, which is known to strongly influence mapping results in metagenomics.
2. The authors state: "For comparison, only the forward reads from HiSeq 2000 were used." One of the strengths of Illumina sequencing is the paired end capability. In principle, this should also be available for the BGISEQ-500 platform. The authors ought to use the forward and reverse reads for their comparison.
3. In their section "Quality control (QC) of sequencing data", the authors mention a new method to quality control sequencing reads. I cannot find an accurate and thorough description of this method. I would also be good to to motivate the need for this method better. This seems to be a key part of the manuscript and should be extensively reworked. It would further be good to provide this method as free and open-source a software for readers to use.
4. The authors ought to explain the challenges in metagenomics sequencing compared to other sequencing application better.

Minor issues:
1. Page 2, line 7: The authors mention that they used the same fecal subjects. It would be good if the authors could specify what exactly this means. Are these samples from the same person? The same piece of stool? Before or after homogenization?
2. Page 5, line 2-4: I don't understand why the authors decided to do the following: "To eliminate the influence of different number of reads per sample in intra or cross-platform analyses, unique mapped reads were downsized to 20 million for each subject." According to Suppl. File 3, all samples have more than 30M sequencing reads. I do not understand the motivation to down sample to 20M reads.

3. The authors mention that GC content and gene abundance are related on page 6. On page 7 they mention that this relationship does not vary between sequencing platforms. The authors need to clarify.

**Level of Interest**

Please indicate how interesting you found the manuscript: An article whose findings are important to those with closely related research interests

**Quality of Written English**

Please indicate the quality of language in the manuscript: Acceptable

**Declaration of Competing Interests**

Please complete a declaration of competing interests, considering the following questions:

- Have you in the past five years received reimbursements, fees, funding, or salary from an organisation that may in any way gain or lose financially from the publication of this manuscript, either now or in the future?
- Do you hold any stocks or shares in an organisation that may in any way gain or lose financially from the publication of this manuscript, either now or in the future?
- Do you hold or are you currently applying for any patents relating to the content of the manuscript?
- Have you received reimbursements, fees, funding, or salary from an organization that holds or has applied for patents relating to the content of the manuscript?
- Do you have any other financial competing interests?
- Do you have any non-financial competing interests in relation to this paper?

If you can answer no to all of the above, write 'I declare that I have no competing interests' below. If your reply is yes to any, please give details below.

I declare that I have no competing interests.

I agree to the open peer review policy of the journal. I understand that my name will be included on my report to the authors and, if the manuscript is accepted for publication, my named report including any attachments I upload will be posted on the website along with the authors' responses. I agree for my report to be made available under an Open Access Creative Commons CC-BY license (http://creativecommons.org/licenses/by/4.0/). I understand that any comments which I do not wish to

be included in my named report can be included as confidential comments to the editors, which will not be published.

I agree to the open peer review policy of the journal

To further support our reviewers, we have joined with Publons, where you can gain additional credit to further highlight your hard work (see: https://publons.com/journal/530/gigascience). On publication of this paper, your review will be automatically added to Publons, you can then choose whether or not to claim your Publons credit. I understand this statement.

Yes
