# Supplementary material for: Assessment of the cPAS-based BGISEQ-500 platform for metagenomic sequencing
Source: Gigascience. 2017 Dec 23;7(3):gix133. doi: 10.1093/gigascience/gix133 (PMC5848809; doi:10.1093/gigascience/gix133)
Supplement: Reviewer_2_Report_(Revision_1) -- Daniel Mende — 06 Dec 2017 Reviewed [file gix133_reviewer_2_report_(revision_1).pdf]

Reviewer Report

Title: Assessment of the cPAS-based BGISEQ-500 platform for metagenomic sequencing

Version: Revision 1 **Date:** 06 Dec 2017

Reviewer name: Daniel Mende

Reviewer Comments to Author:

I would like to thank the authors for addressing most of my concerns. I'm especially happy the addition of the HiSeq 4000 sequencing data, even though the HiSeq 2500 would have been preferable due to its longer read length.

While the authors attempted to address my concern about read length, I am not fully satisfied with their solution. In my opinion the authors should have added a comparison to the longer reads produced by Illumina HiSeq technology (up to 250bp read length). As the authors state themselves: "We believe that the future rapid updates of sequencing techniques would soon provide longer and even more accurate reads and improve the accuracy for reads-based metagenomic taxonomic and functional annotation and quantification." But this (partially) already reality and it would be fair to compare the new BGISEQ-500 platform to the newest Illumina technologies. By using 100bp reads across the board, Illumina results might be penalized while BGISEQ-500 uses its full read length.
I don't think it's necessary to add more analyses for this, but it would be good to discuss this (i.e. "future rapid updates" have already happened).

Level of Interest

Please indicate how interesting you found the manuscript: An article whose findings are important to those with closely related research interests

Quality of Written English

Please indicate the quality of language in the manuscript: Acceptable

Declaration of Competing Interests

Please complete a declaration of competing interests, considering the following questions:

- Have you in the past five years received reimbursements, fees, funding, or salary from an organisation that may in any way gain or lose financially from the publication of this manuscript, either now or in the future?

- Do you hold any stocks or shares in an organisation that may in any way gain or lose financially from the publication of this manuscript, either now or in the future?
- Do you hold or are you currently applying for any patents relating to the content of the manuscript?
- Have you received reimbursements, fees, funding, or salary from an organization that holds or has applied for patents relating to the content of the manuscript?
- Do you have any other financial competing interests?
- Do you have any non-financial competing interests in relation to this paper?

If you can answer no to all of the above, write 'I declare that I have no competing interests' below. If your reply is yes to any, please give details below.

I declare that I have no competing interests

I agree to the open peer review policy of the journal. I understand that my name will be included on my report to the authors and, if the manuscript is accepted for publication, my named report including any attachments I upload will be posted on the website along with the authors' responses. I agree for my report to be made available under an Open Access Creative Commons CC-BY license (http://creativecommons.org/licenses/by/4.0/). I understand that any comments which I do not wish to be included in my named report can be included as confidential comments to the editors, which will not be published.

I agree to the open peer review policy of the journal

To further support our reviewers, we have joined with Publons, where you can gain additional credit to further highlight your hard work (see: https://publons.com/journal/530/gigascience). On publication of this paper, your review will be automatically added to Publons, you can then choose whether or not to claim your Publons credit. I understand this statement.

Yes
