# Supplementary material for: Assessment of the cPAS-based BGISEQ-500 platform for metagenomic sequencing
Source: Gigascience. 2017 Dec 23;7(3):gix133. doi: 10.1093/gigascience/gix133 (PMC5848809; doi:10.1093/gigascience/gix133)
Supplement: Supplemental material [file Rebuttal Figure 1.pdf › Rebuttal Figure 1.pdf]

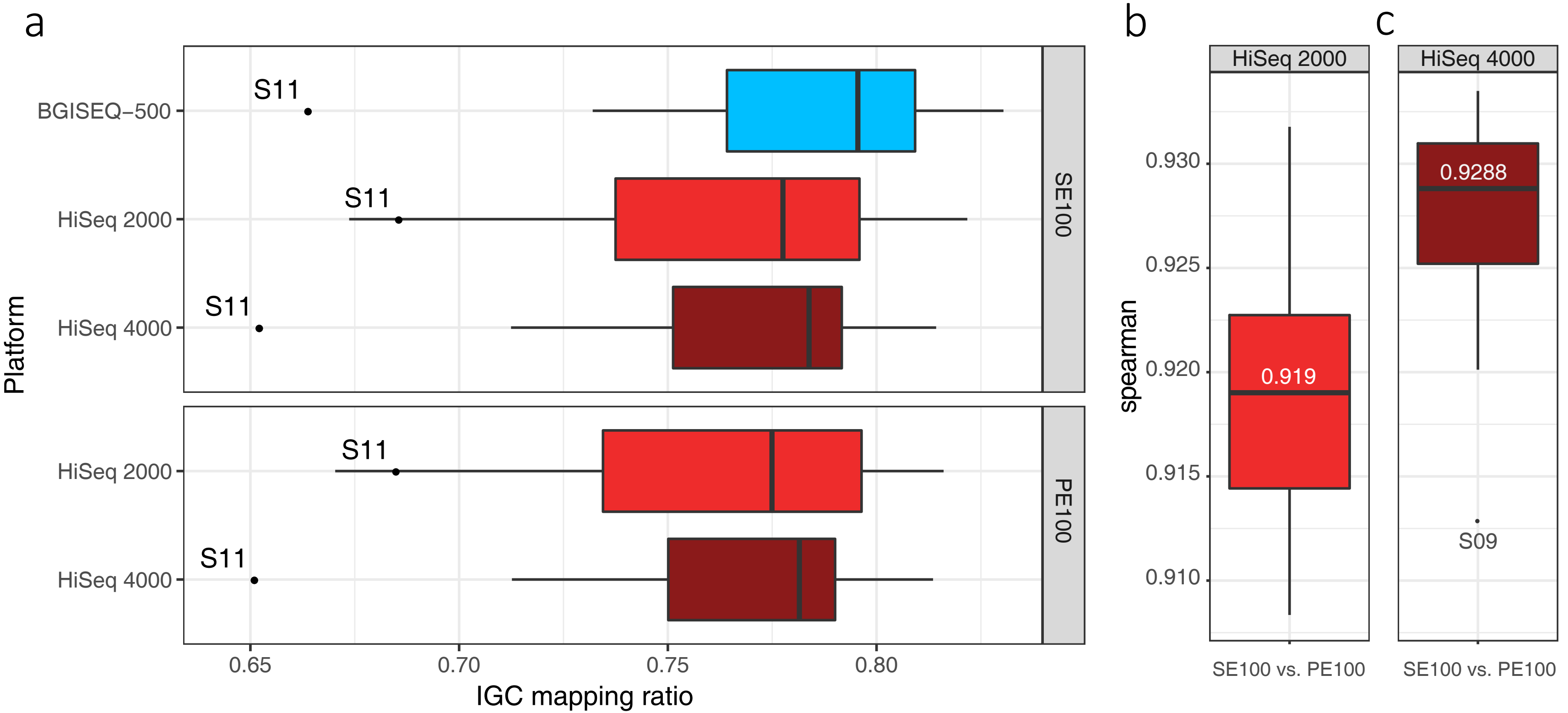
a
b
c
Platform
BGISEQ−500
HiSeq 2000
HiSeq 4000
HiSeq 2000
HiSeq 4000
S11
S11
S11
S11
S11
SE100
PE100
0.65
0.70
0.75
0.80
IGC mapping ratio
HiSeq 2000
HiSeq 4000
spearman
0.930
0.925
0.920
0.915
0.910
0.919
0.9288
S09
SE100 vs. PE100
SE100 vs. PE100
