# Supplementary material for: Assessment of the cPAS-based BGISEQ-500 platform for metagenomic sequencing
Source: Gigascience. 2017 Dec 23;7(3):gix133. doi: 10.1093/gigascience/gix133 (PMC5848809; doi:10.1093/gigascience/gix133)
Supplement: Supplemental material [file gix133_supp.zip › Supplementary Method.doc]

Supplementary Method

For

Assessment of the cPAS-based BGISEQ-500 platform for metagenomic sequencing

**Overall Accuracy (OA) control strategy**

Considering that a routinely tail trimming QC pipeline would not be sensitive to detect and filter reads with randomly distributed low-quality bases, we developed an overall accuracy (OA) based QC method **(Additional file 2)**.

Briefly, this QC method contain following steps.

**We firstly calculate the accuracy probabilities of each base** **using the following equations:**

1) Q = -10 log_10_ E
2) P = 1 - E

Where *Q* is the Phred quality score of each base, *E* is the error probability of each base.

**Then we calculate the overall accuracy probability (OA) of each read using the following equation:**

Where *P* is the accuracy per base, *n* is the read length, *i* indicates a specific base position from the 5’ to the 3’ end, and *m* indicates the number of bases with lowest accuracy to be ignored during accuracy calculation.

For a read X with length of 10 bp, in which 9 bases have accuracy values of 99.9% (*Q* = 30) and 1 base *i* has accuracy of 90% (*Q =* 20), and if *m* is set to 1, then base *i* of a lowest accuracy value of 90% would not be included in the overall accuracy calculation for read X, and the OA of read X would be 91.35% (0.99 ^9). If we set *m* to 0, all bases would be used and the OA of read X would be 89.1% (0.99^9 * 0.9).

For each read, an initial 30-mer seed sequence is selected at the 5’ end of the read and its overall accuracy, defined as OA_seed_, is calculated. To ensure high data quality, OA_seed_ is defined as 0.9 using *m* equal to 0 with zero low quality bases allowed. Once the seed position of the read has been defined, the seed would extend to keep the longest contiguous read fragment in which the OA, defined as OA_fragment_, is above a defined accuracy threshold. In this study, we set OA_fragment_ equal to or greater than 0.8 using *m* equal to 1.

The Perl scripts for overall accuracy based QC pipeline are freely available for download and reuse from GigaDB (XX) and Github (https://github.com/Scelta/OAFilter).

Data analysis and statistics—assessing intra- and inter- platform consistency.

To ensure a robust estimate representative of the quantitative performance, a variety of tools/pipelines were applied to process and compare metagenomic datasets from intra-technical and cross-platform replications. One subject was excluded from the analysis (S01, **Additional file 4**) because of the low IGC mapping ratio of the HiSeq 2000 dataset.

**Estimation of random fluctuation in mapped reads number of gene**

To infer reasonable gene occurrence in duplicate experiments, we first assessed the expected read counts random fluctuations based on the Bayesian method described by Acuic and Claverie [1].

The estimation equation is shown as follows:

Where is the read counts of a given gene observed in the first replicate, and is the read counts observed in the second replicate.

The probability of *y* falling within was summed as the confidence interval (CI), at the significance level. Here we use 99% CI and significance at the 0.01 level (. The y values falling outside the [y_min_, y_max_]_0.01_ interval represent most unlikely reads with random fluctuating between the two replicates, which may be introduced by other factors.

Based on the equation, when a given gene was supported by 10 mapped reads (x = 10), in the corresponding replicate, it could be detected by at least 1 mapped read (y_min_ >= 1) with a probability of 99% (dashed line, **Fig. 2a, Fig. 3a**).

Therefore, in order to precisely assess the intra-platform reproducibility and inter-platform consistency, we considered the genes which were identified by 10 or more reads as highly-reproducible (HR) genes and used them when we performed difference quantification, correlation, and statistical analysis.

**Quantifying the differences of gene abundances between replicates or paired-group**

The area left of the cumulative distribution curve (ALC) values were used to quantify the mean differences of gene relative abundance between two replicates or two paired groups as described previously [2].

We calculated the absolute difference of relative abundance of HR genes between replicates under the two different situations. The upper boundary of the ALC value was determined based on all observed differences including those likely caused by random fluctuations during read counting. The lower boundary of the ALC value was determined by ignoring all the differences likely caused by random fluctuations (fluctuations within 99% CI). The cumulative distributions of replicate differences were plotted (**Additional file 5**). In this study, we reported the replicate gene relative differences with a range between lower and upper ALC values.

**Statistics** **tests on high reproducible genes**

Due to the discrete nature of metagenomic data, we chose HR genes present in at least 6 pairs of replicates to ensure necessary power for the statistical tests. Shapiro-Wilk tests were first conducted to assess the normality of relative abundance distributions for these genes. For genes with normal distribution (Shapiro-Wilk test, P > 0.05), paired t-tests were used. For genes that were not normally distributed, paired sign tests were conducted. The P values were Benjamini-Hochberg adjusted, with a FDR of 5% considered as significant. All tests were performed in R (Version 3.3.3).

**Robust linear** **regression analysis for abundance of genes and their GC content**

Robust linear regression analysis was conducted to assess the relationship between abundance of genes from a species and their GC-content (lmrob () in R robustbase package, version 0.92-7). The top 20 species with genes exhibiting the most significant differences in abundance between the two platforms were used for this evaluation **(Additional file 6)**. Before analysis, we first normalized the relative abundance of genes from each species, excluding genes with extreme GC content (top 0.5% value and bottom 0.5% value).

In each sample, two robust linear models were built for each of the 20 species. One model was based on the genes exhibiting the most significant differences in abundance between the two platforms and their GC content **(Fig.4c, Additional file 7),** and the other was based on all HR genes selected for testing from these species and their GC content **(Fig.4d, Additional file 7)**.

**Generalized linear model (GLM) regression analysis for relative species abundance and their GC content across two platforms**

Generalized linear model (GLM) regression was used to assess whether the association between relative abundance and GC content varied between the BGISEQ-500 and the HiSeq 2000 sequencing platforms. Samples were categorized into eight categories based on abundance (high or low), GC content (high or low), and sequencing platform (BGISEQ-500 or HiSeq 2000). High relative abundance and GC content were classified as being above the median level (abundance median = 0.2844, GC content median = 43.8%). A log linear model was used to model the expected number of samples in each of the 8 categories. Let X, Y, and Z be random variables describing GC content, relative abundance, and sequencing platform for each sample:

X ∼ GC content (0=below median, 1=above median), *i*=0/1

Y ∼ Abundance (0=below median, 1=above median), *j*=0/1

Z ∼ Sequencing (0=BGISEQ-500, 1=HiSeq 2000), *k*=0/1

Then, the probability of being in category ijk is denoted as:

and the expected number of counts in category ijk is

where n is the total sample size.

Two models were used to model expected counts. The first (model 1) models the null hypothesis that the association between relative abundance and GC content does not vary across sequencing platform. The second (model 2) represents the alternative hypothesis that the association between relative abundance and GC content vary across sequencing platform.

Model 1:

Model 2:

A likelihood ratio test was used to test the significance of the addition of the three-way interaction term (). A significant test statistic would suggest that the null hypothesis that the association between relative abundance and GC content does not vary across the BGISEQ-500 and the HiSeq 2000 sequencing platforms can be rejected.

**Reference**

1. Audic S, Claverie JM. The significance of digital gene expression profiles. Genome Res. 1997;7:986–95.

2. Mestdagh P, Hartmann N, Baeriswyl L, Andreasen D, Bernard N, Chen C, et al. Evaluation of quantitative miRNA expression platforms in the microRNA quality control (miRQC) study. Nat. Methods. 2014;11:809–15.
